# Ameliorative Effect of Erjing Pills on Retinal Damage in Rats with Diabetic Retinopathy

**DOI:** 10.3390/ph19060940

**Published:** 2026-06-15

**Authors:** Xiangduo Zuo, Mijia Mei, Yiping Wang, Meixia Wang, Xiaolan Liu, Xiang Xu, Yirong Ni, Jingping Li

**Affiliations:** 1 School of Chinese Materia Medica and Yunnan Key Laboratory of Southern Medicinal Resource, Yunnan University of Chinese Medicine, 1076 Yuhua Road, Kunming 650500, China; zxd15265935653@163.com (X.Z.); 15559803641@163.com (M.M.); 15253145309@163.com (M.W.); 15125888047@163.com (X.L.); xxrrqqcn123@163.com (X.X.); 2 School of Pharmaceutical Science and Yunnan Key Laboratory of Pharmacology for Natural Products, Kunming Medical University, Kunming 650500, China; 3 Beijing Ocean Peace Environmental Protection Technology Co., Beijing 100024, China; xuhongju91@163.com; 4 Office of International Exchange & Cooperation, South China Normal University Guangzhou Shipai Campus, No. 55, West Zhongshan Avenue, Tianhe District, Guangzhou 510631, China

**Keywords:** diabetic retinopathy (DR), Erjing Pills (EJPs), brownNorway (BN) rats, traditional Chinese medicine, AGE-RAGE signaling

## Abstract

**Background**: Diabetic retinopathy (DR) is one of the major complications of diabetes mellitus. EJPs (Erjing Pills) are believed in Traditional Chinese Medicine to have the effects of a nourishing essence and a brightening of the eyes, but the specific effect on DR remains unclear. This study aims to investigate the therapeutic effects and underlying mechanisms of EJPs on DR. **Methods**: The chemical profile of EJPs was characterized by UHPLC-MS. Network pharmacology and molecular docking were employed to predict its active ingredients and potential targets. A DR rat model was induced by streptozotocin. Retinal morphology and function were assessed by OCT, FFA, and H&E staining. The expressions of proteins and mRNAs related to the AGE-RAGE pathway, oxidative stress, inflammation, and tight junctions were detected by Western blot, qPCR, and ELISA. **Results**: LC-MS and network pharmacology analysis identified 638 common targets between EJPs and DR, with core targets including SRC, AKT1, and MAPK1, primarily enriched in the AGE-RAGE signaling pathway. Molecular docking confirmed strong binding (binding energy < −5.0 kcal/mol) between key EJP constituents and core targets. In vivo, EJP treatment significantly alleviated retinal vascular leakage, improved retinal thickness, and alleviated histopathological damage. In addition, EJPs downregulated the AGEs-RAGE/NF-κB axis and pro-inflammatory cytokines while enhancing antioxidant defenses and tight junction proteins in the retinas of DR rats. **Conclusions**: EJPs ameliorate DR by protecting the blood–retinal barrier and modulating the AGE-RAGE/oxidative stress/inflammation network, demonstrating a multi-component, multi-target, and multi-pathway mechanism. This study provides a mechanistic basis for the potential application of EJPs in DR management.

## 1. Introduction

Diabetic retinopathy (DR) is one of the main complications of diabetes and also one of the main causes of visual impairment and blindness in patients [1]. In recent years, research has gradually revealed the complex pathological mechanisms of DR. Diabetic retinal neurodegeneration (DRN) is an early event in the pathogenesis of DR and is closely related to the occurrence and development of microvascular abnormalities. DR is not simply a microvascular disease but a highly tissue-specific neurovascular complication. DRN combines retinal neurodegeneration with pathological changes in the vascular system, namely impaired neurovascular unit (NVU) function in the retina [2,3]. The disorder of the NVU structure and function is an early event of DR and directly mediates the further development of DR [4,5,6]. Therefore, early identification of the pathological features of DR and taking effective prevention and treatment measures are crucial for reducing the risk of irreversible visual impairment in patients.

*Polygonatum sibiricum* (Chinese: “huang jing”) and *Lycium barbarum* L. (Chinese: “gou qi”) constitute a classic herbal pair, documented as early as the Northern Song Dynasty in “Shengji Zonglu”. This combination, typically in a 1:1 ratio and referred to as Erjing Pills (EJPs), has been historically used to alleviate a cluster of symptoms, including blurred vision, tinnitus, dizziness, polydipsia (excessive thirst), and lumbar weakness. Modern pharmacological investigations have revealed that the individual herbs [7,8] *Polygonatum sibiricum* [9,10] and *Lycium barbarum* L. [11,12] possess a range of bioactivities relevant to metabolic and inflammatory disorders, such as hypoglycemic, antioxidant, anti-inflammatory, anti-aging, and neuroprotective effects. While several studies have explored the multifaceted potential of the EJP formulation itself [13,14,15,16], its specific mechanisms of action against diabetic retinopathy (DR) remain unclear. Our preliminary in vitro studies on retinal pigment epithelium (RPE) cells indicated that EJP treatment significantly reduced the levels of key DR-related pathogenic factors, including reactive oxygen species (ROS), vascular endothelial growth factor (VEGF), and intercellular adhesion molecule-1 (ICAM-1). This prompted us to further investigate its therapeutic potential and mechanisms in DR.

In the setting of diabetes mellitus (DM), non-enzymatic glycation is markedly accelerated, resulting in the accumulation of its products, advanced glycation end products (AGEs), in circulation and retinal tissues. Upon binding to their receptor (RAGE), AGEs generate ROS and reactive nitrogen species (RNS), thereby inducing oxidative stress. The excessive production of ROS and RNS, in turn, promotes further AGE formation, establishing a vicious cycle that continuously damages retinal neurons, glial cells, and microvascular endothelial cells [17,18]. Concurrently, the endogenous antioxidant defense system becomes dysregulated. In particular, nuclear factor E2-related factor 2 (Nrf2) exhibits impaired nuclear translocation and transcriptional activity, leading to the downregulation of its downstream antioxidant target genes, such as heme oxygenase-1 (HO-1), superoxide dismutase (SOD), and glutamate-cysteine ligase (GCL). This results in decreased glutathione (GSH) synthesis and reduced antioxidant enzyme activities, thereby compromising the capacity to scavenge and defend against oxidative stress [19]. Oxidative stress further activates nuclear factor-kappaB (NF-κB), which stimulates glial cells to release a variety of inflammatory factors, including interleukin-6 (IL-6), tumor necrosis factor-alpha (TNF-α), interleukin-1beta (IL-1β), and ICAM-1 [20]. This perpetuates a chronic inflammatory state in the retina, ultimately leading to DRN, characterized predominantly by neuronal apoptosis and further promoting the initiation and progression of retinal microvascular disease [21].

The purpose of this study is to explore the mechanism of EJPs in preventing and treating DR by improving damage to retinal neurovascular unit function in diabetes through network pharmacology and animal experiments, so as to provide a valuable scientific basis and reference for the application of EJPs in preventing and treating diabetes and its retinopathy.

## 2. Results

### 2.1. Chemical Composition of EJPs

**Figure 1:**
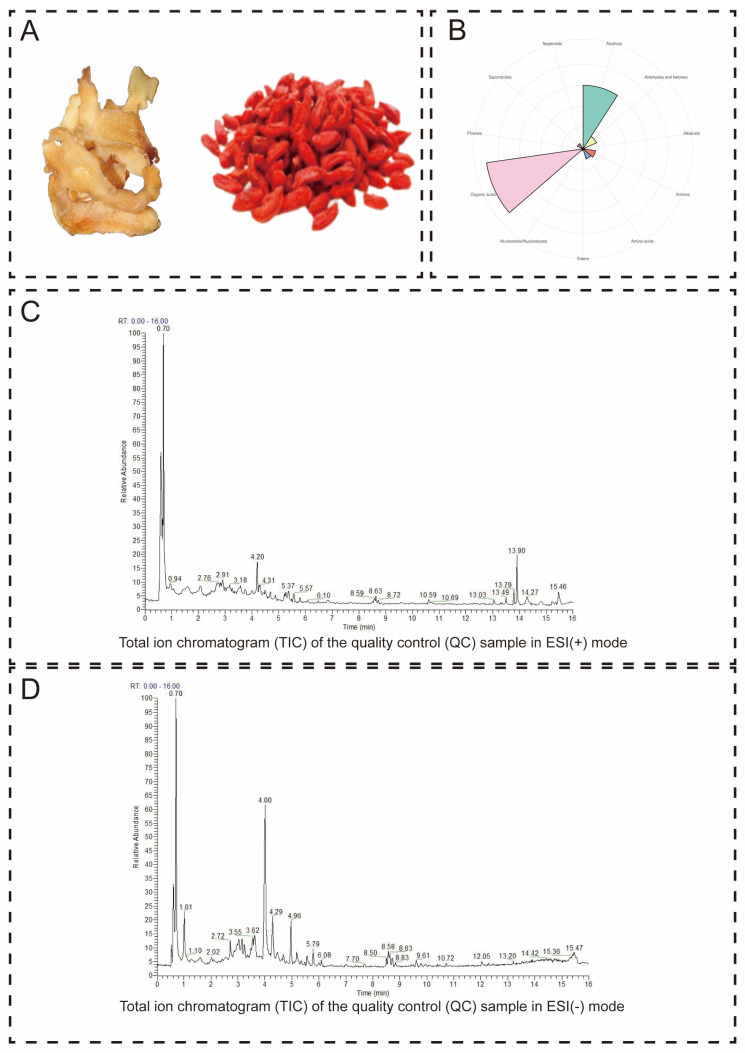
Metabolomic profiling of EJPs extract. (**A**) Photographs of the raw medicinal materials used in the EJPs formula. (**B**) Relative abundance distribution of detected metabolite classes, categorized by chemical taxonomy. (**C**) Total ion chromatogram (TIC) of the quality control (QC) sample in positive electrospray ionization (ESI+) mode. (**D**) Total ion chromatogram (TIC) of the QC sample in negative electrospray ionization (ESI−) mode.

EJPs were prepared by decocting *Polygonatum sibiricum* and *Lycium barbarum* L., followed by sample preparation for LC-MS analysis (Figure 1A). The mass spectrometric data of EJPs were acquired using a UHPLC-Q Exactive system in both positive and negative ionization modes (Figure 1C,D). A total of 408 components were found in EJPs (Table S1), with the following primary classes: Organic acids (26.9%), Alcohols (17.4%), Aldehydes and ketones (4.2%), Amino acids (2.7%), Amines (3.2%), Carbohydrates (1.5%) (Figure 1B).

### 2.2. Network Pharmacology Analysis of EJPs in DR

**Figure 2:**
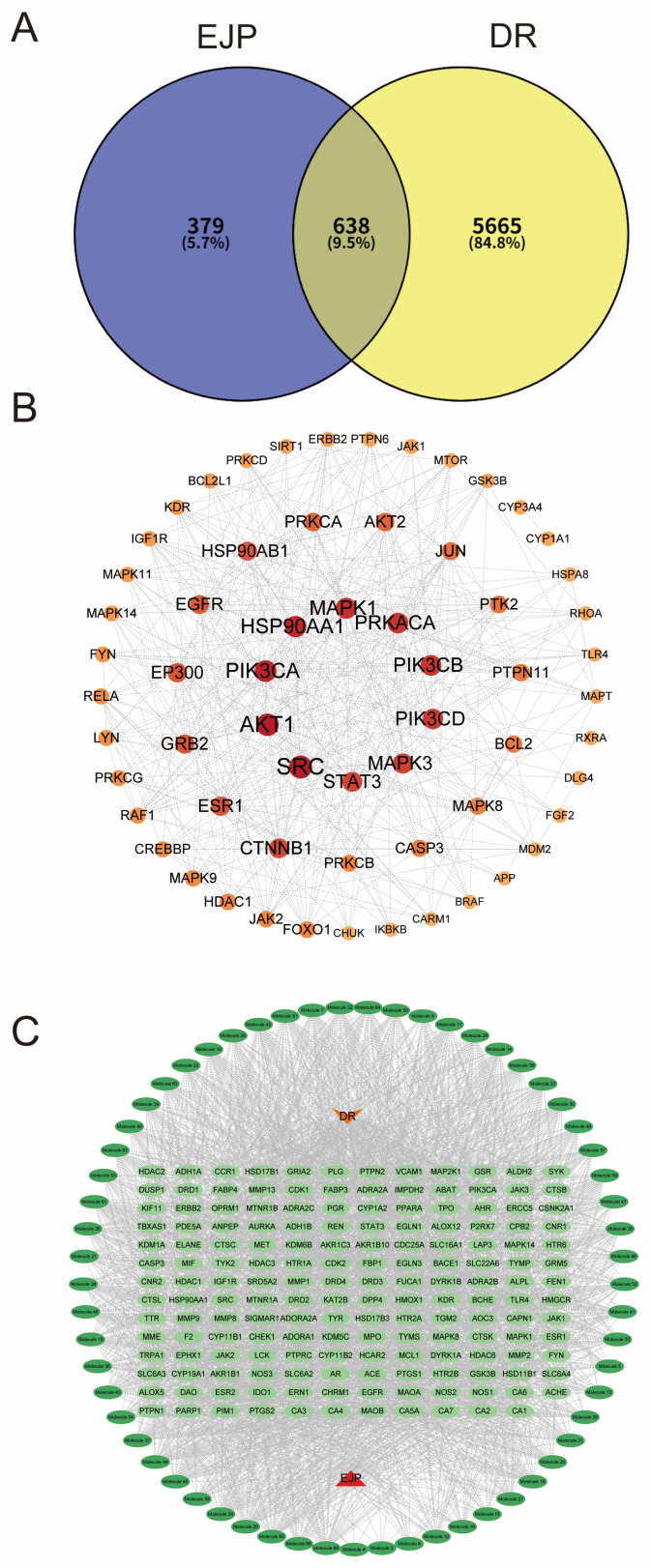
The network pharmacology analysis of the EJPs in DR. (**A**) Overlapping targets between EJPs and DR. (**B**) Drug–constituent–target–disease network relationships. (**C**) Protein–protein interaction (PPI) network.

Based on the 408 components identified, the potential bioactive ingredients in EJPs were further screened using the SwissADME database, with criteria of high gastrointestinal absorption and drug-likeness. This process identified 66 major active ingredients in EJPs, targeting 1017 potential targets in EJPs, while 6303 targets associated with DR were recovered. A Venn analysis showed that 638 targets overlapped (Figure 2A). To identify the key targets, an EJP–component–target–disease interaction network was constructed (Figure 2B). Subsequent protein–protein interaction (PPI) analysis of the network targets identified SRC, AKT1, PIK3CA, HSP90AA1, MAPK1, PRKACA, PIK3CB, PIK3CD, MAPK3, and STAT3 as the top-ranked hub proteins (Figure 2C).

### 2.3. Results of GO Functional Analysis and KEGG Enrichment Analysis

**Figure 3:**
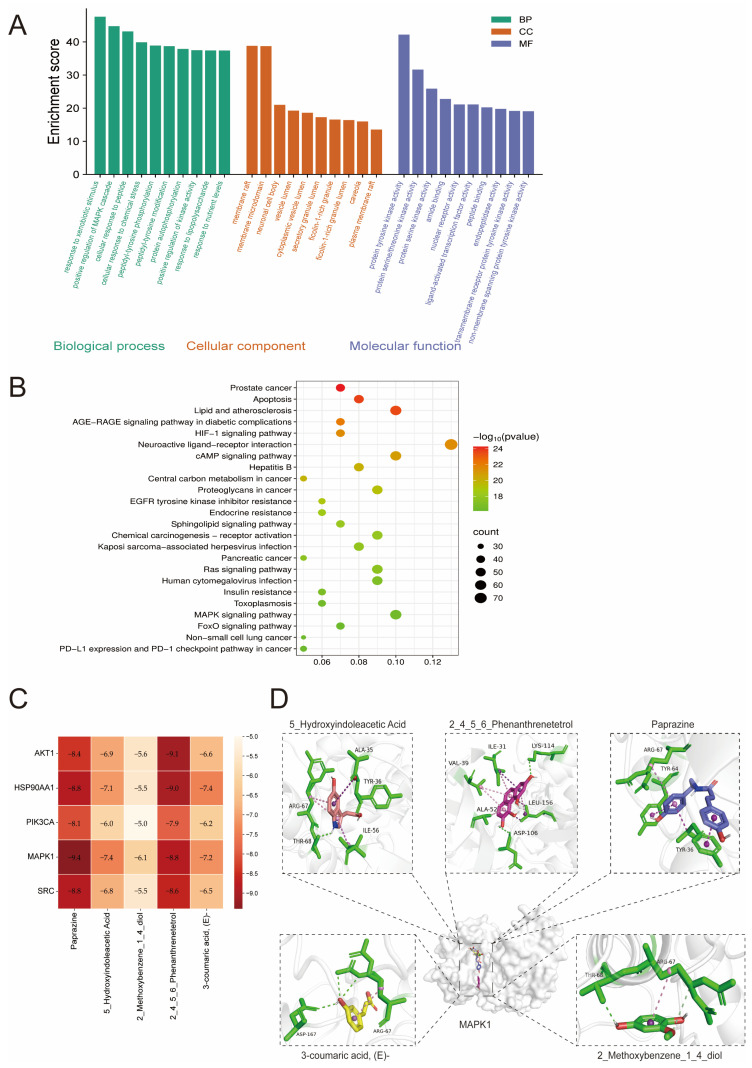
GO, KEGG enrichment analysis, and molecular docking. (**A**) GO enrichment analysis, top 10 entries. (**B**) KEGG pathway enrichment analysis, top 25 entries. (**C**) Heat map of binding energy between active ingredients and target hub proteins, molecule-target binding energies, all below −5 kcal/mol. (**D**) Visual analysis of docking results between EJPs and DR core target molecules. Different colors in stick models represent various ligand molecules as indicated above.

To gain insights into the functions of the potential targets, Gene Ontology (GO) and Kyoto Encyclopedia of Genes and Genomes (KEGG) enrichment analyses were performed. The GO analysis covers three domains: biological process (BP), cellular component (CC), and molecular function (MF). The BP analysis indicated that the response to xenobiotic stimulus and the regulation of the MAPK cascade may play crucial roles in the therapeutic effect of EJPs on DR (Figure 3A). The MF terms were primarily associated with protein tyrosine kinase activity, protein serine/threonine kinase activity, and amide binding. Additionally, CC primarily consisted of the membrane raft, the membrane microdomain, and the neuronal cell body. Among the 25 KEGG pathways (*p* < 0.05), the AGE-RAGE signaling pathway in diabetic complications and the MAPK signaling pathway were the primary focus (Figure 3B).

### 2.4. Result of Molecular Docking

Based on the protein-protein interaction (PPI) network analysis, the top five core targets (ranked by degree value) were selected for molecular docking. Docking simulations between these key targets and the five main chemical constituents of EJPs were performed using AutoDock Vina 1.5.7 to calculate the binding affinities, with the results visualized in Figure 3C. Paprazine, 5-Hydroxyindoleacetic Acid, 2-Methoxybenzene-1,4-diol, 2,4,5,6-Phenanthrenetetrol, and 3-coumaric acid, (E)-displayed binding energies of less than −5.0 kcal/mol when docked with AKT1, HSP90AA1, PIK3CA, MAPK1, and SRC proteins, respectively. These findings indicated that the modulatory effects of these five compounds on AKT1, HSP90AA1, PIK3CA, MAPK1, and SRC proteins play a critical role in the treatment of diabetic retinopathy (DR). The results of the visual analysis are illustrated in Figure 3D.

### 2.5. EJPs Might Ameliorative Retinal Damage in Diabetic Rats

**Figure 4:**
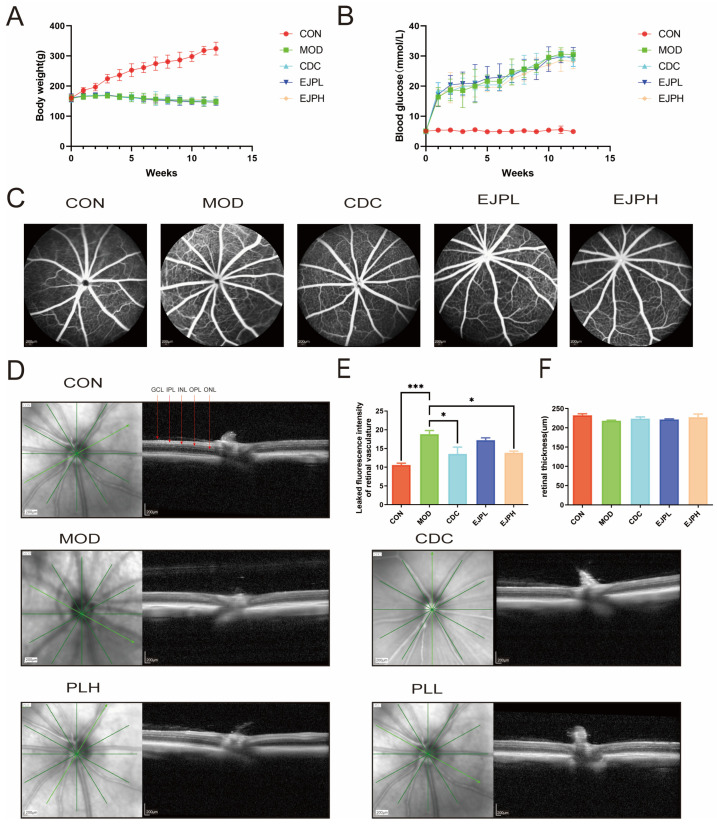
The effects of EJPs on body weight, blood glucose levels, and retinal damage in diabetic rats. (**A**) Changes in body weight in DR rats, *n* = 6. (**B**) Blood glucose changes in DR rats, *n* = 6. (**C**) Representative images of sodium fluorescein (white) in the retina of rats in each group. Alterations in fluorescein sodium leakage from the retinal microvasculature detected by fluorescein fundus angiography in various groups of rats. The white part of the figure shows the distribution and leakage points of sodium fluorescein in retinal blood vessels. Scale bar indicates 200 μm. *n* = 3. (**D**) Representative images of retinal thickness in each group of rats. Scale bar indicates 200 μm. *n* = 3. (**E**) Quantification of fluorescein sodium intensity in the retina in each group. (**F**) Retinal thickness measurement results of rats in each group. The red arrows are GCL: ganglion cell layer; IPL: inner reticular layer; INL: inner nuclear layer; OPL: outer reticular layer; ONL: outer nuclear layer. Data are expressed as mean ± SD. Scale bar indicates 200 μm. * *p* < 0.05, *** *p* < 0.001.

**Figure 5:**
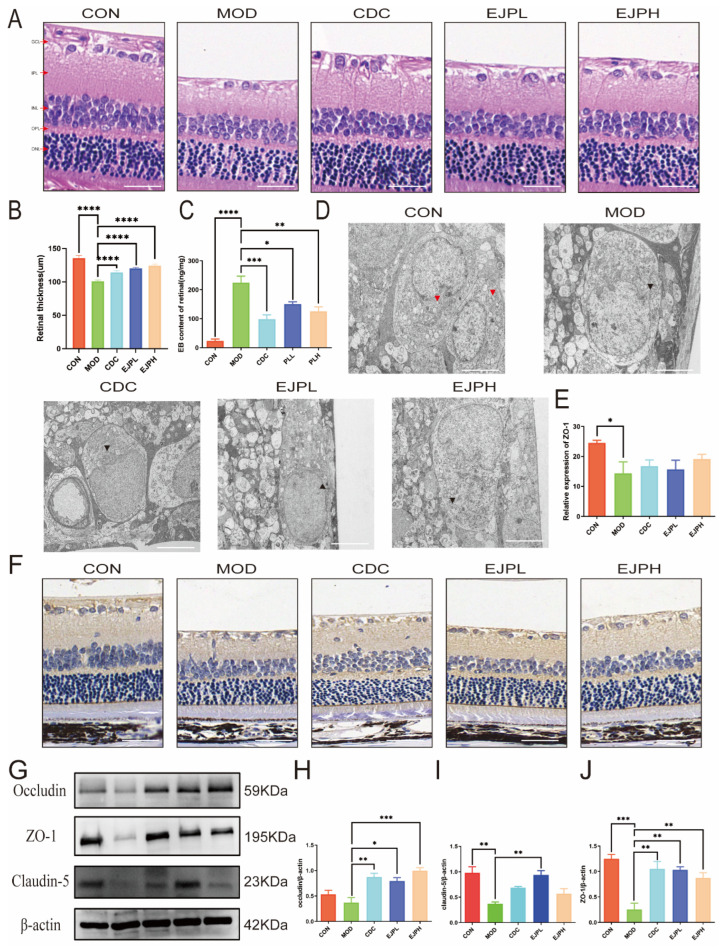
The effects of EJPs on retinal tissue in diabetic rats and on the expression of tight junction proteins in retinal tissue. (**A**) Alterations in the histopathological morphology of the retina in each group of rats observed by HE staining. The black arrows indicate that the thickness of each layer of the retina in diabetic rats is thinner, the number of ganglion cells is reduced, and the arrangement of cells in each layer is disordered. Scale bar indicates 25 μm. *n* = 3. (**B**) Statistical analysis of the changes in retinal thickness in each group based on HE staining results. (**C**) Results of Evans blue leakage testing of retinal tissue. (**D**) Alterations in RGCs in each group of rats as observed by transmission electron microscopy. Red and black triangles are used to respectively represent normal and atrophied mitochondria. Scale bar indicates 5 μm. *n* = 3. (**E**) Quantification of ZO-1 protein intensity in retinal tissue of rats in each group. (**F**) ZO-1 protein expression in the retinal tissue of rats in each group was detected by immunohistochemical staining. The positive expression of immunohistochemical staining markers is brown. The cell nuclei were stained with hematoxylin and were blue-purple. Scale bar indicates 25 μm. *n* = 3. (**G**) The expression of occludin, claudin-5 and ZO-1 proteins in the retinal tissues of rats in each group was detected by Western blot. *n* = 3. (**H**–**J**) Bar chart of occludin, claudin-5 and ZO-1 protein expression. Mean ± SD. * *p* < 0.05, ** *p* < 0.01, *** *p* < 0.001, **** *p* < 0.0001.

We first established an animal model of diabetic rats; the modeled rats exhibited typical characteristics of high blood sugar and impaired retinal structure and function. During the experiment, the weight changes of each group of rats were recorded weekly. Compared with the control group, the weight of the model group rats significantly decreased, while there was no significant difference in weight changes between each treatment group and the model group (Figure 4A). Weekly monitoring of blood glucose changes in rats showed a significant increase in blood glucose levels in the model group, indicating successful modeling. There was no significant difference in blood glucose changes between the treatment groups and the model group (Figure 4B). To further validate the performance of this model and the efficacy of EJPs, we conducted OCT and FFA examinations to assess changes in the rat retina. The distribution of retinal blood vessels in the normal group of rats is uniform, with no obvious leakage points of fluorescein sodium. The leakage points of fluorescein sodium in the retina of the model group significantly increased (Figure 4C). Compared with the model group, the high-dose EJP group showed a reduction in fluorescein sodium leakage points, suggesting that EJPs have a protective effect on damage to the retinal vascular system (Figure 4E). The retinal thickness of the model group was thinner than that of the normal group, but there was no significant difference (Figure 4D,F). This in vivo observation was corroborated by a subsequent quantitative histomorphometric analysis of retinal thickness (Figure 5B), suggesting a potential restorative effect of EJPs on retinal morphology.

### 2.6. The Effects of EJPs on Histopathology and the Expression of Related Proteins in Rats with Diabetic Retinopathy

To assess specific changes in the cells of the various retinal layers and detect leakage, H&E staining, Evans blue (EB) staining, and transmission electron microscopy were performed. The layers of the retina in the normal group were clear and intact, and the cells were arranged neatly. The model group suffered severe damage to all layers of the retina, with disordered cell arrangement. H&E staining revealed that EJP treatment, particularly at the high dose, ameliorated the disorganized architecture and reduced vacuolization across retinal layers compared to the MOD group. This suggests a potential improvement in overall retinal histopathology (Figure 5A). In addition, we performed morphometric measurements of total retinal thickness on H&E-stained sections using ImageJ 1.54 software. Consistent with the OCT data, quantitative analysis confirmed a significant reduction in retinal thickness in the MOD group compared to the CON group. Critically, treatment with EJPH significantly restored retinal thickness (Figure 5B). The leakage of EB in the retinal tissue of the model group rats was higher than that of the normal group. Compared with the model group, the high-dose EJP group saw a reduction in the amount of EB leakage in rat retinal tissue, further confirming the protective effect of EJPs on retinal vascular permeability (Figure 5C). In the CON group, RGCs exhibited a clear structure, uniform cytoplasm, and normal organelle morphology. In the MOD group, marked pathological changes were observed, including sparse chromatin in the nuclei, vacuolated cytoplasm, reduced and swollen mitochondria, shortened or absent cristae, and irregular organelle morphology. TEM observations indicated a trend towards improved cellular ultrastructure in the treatment groups compared to the MOD group (Figure 5D). Compared with the CON group, ZO-1 expression levels in the retinas of rats in the MOD group were reduced. The treatment group showed an upward trend, but no significant difference was observed (Figure 5E, F). The junctional connections in retinal barrier cells primarily regulate the movement of fluids and molecules through junctional proteins such as occludin, claudin, and ZO-1, thereby maintaining normal retinal function; therefore, we performed WB analysis. The protein levels of ZO-1, occludin, and claudin-5 in the retinas of the model group rats were reduced. After EJP treatment, the expression of these proteins significantly increased, suggesting that the upregulation of these tight junction proteins by EJPs may contribute to the enhancement of retinal barrier function observed in the vascular leakage assays (Figure 5G–J).

### 2.7. The Effects of EJPs on AGE-RAGE and Oxidative Stress in Rats with Diabetic Retinopathy

**Figure 6:**
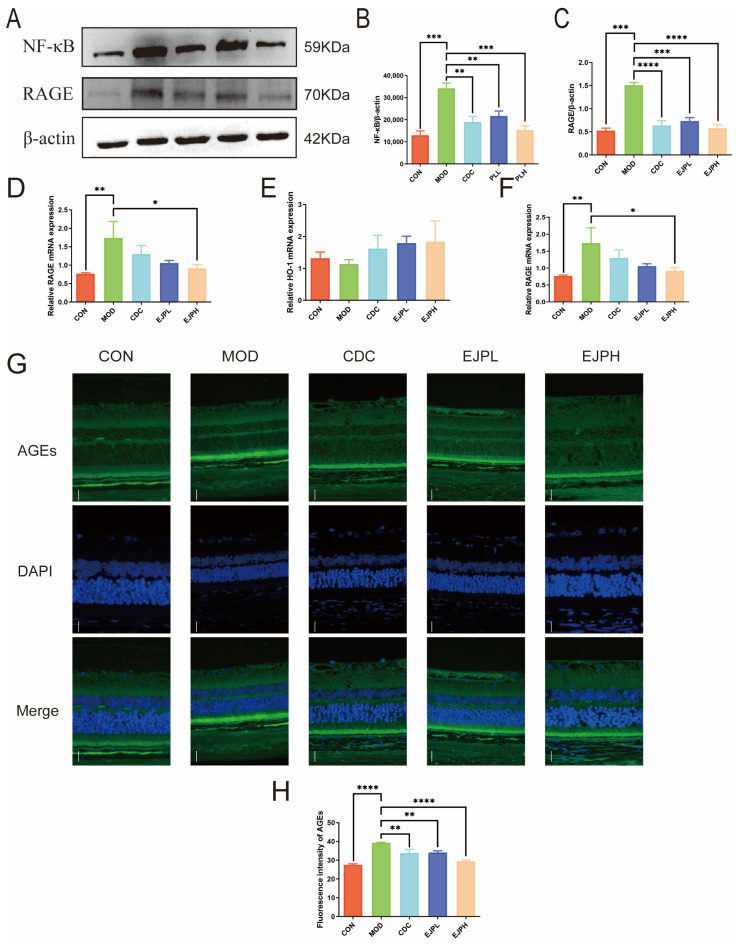
The effects of EJPs on the AGE-RAGE pathway. (**A**) The expression of AGEs and RAGE proteins in the retinal tissues of rats in each group was detected by Western blot. *n* = 3. (**B**) Bar chart of NF-κB protein expression. (**C**) Bar chart of RAGE protein expression. (**D**–**F**) The expression of Nrf2, HO-1, and RAGE mRNA in the retinal tissues of rats in each group was detected by RT-qPCR. (**G**) Immunofluorescence analysis of AGE distribution in rat retinal tissue. Scale bar indicates 25 μm. *n* = 3. (**H**) Statistical analysis of expression levels across groups. Mean ± SD. * *p* < 0.05, ** *p* < 0.01, *** *p* < 0.001, **** *p* < 0.0001.

AGE-RAGE, by activating the NF-κB pathway, interacts with the Nrf2 antioxidant pathway to form a cross-inhibitory relationship; these are two key pathways that are dysregulated in diabetes-related diseases. Western blot analysis showed that the protein levels of RAGE and NF-κB were significantly elevated in the retinas of rats in the MOD group compared to the CON group (Figure 6A–C). In contrast, EJP treatment significantly attenuated these increases, with the high-dose group showing extremely significant inhibition compared to the MOD group (Figure 6A–C).

In addition, compared with the CON group, *RAGE* mRNA expression was significantly elevated in the MOD group (Figure 6D); compared with the MOD group, the EJPH groups significantly reduced *RAGE* mRNA expression (Figure 6D). Furthermore, compared with the CON group, HO-1 expression was reduced in the MOD group, but the difference was not statistically significant. Compared with the MOD group, mRNA expression levels in the treatment groups showed an upward trend. Nrf2 mRNA expression was significantly reduced in the MOD group. Compared with the MOD group, the CDC and EJPH group significantly increased their mRNA expression (Figure 6E,F). At 12 weeks post-treatment, green immunofluorescence staining of retinal AGEs in rats revealed that AGEs were expressed throughout all layers of the retina. ImageJ analysis of fluorescence intensity indicated that the fluorescence intensity of AGEs in the treatment group was lower than that in the MOD group (Figure 6G,H). Taken together, these data indicate that EJP treatment is associated with the modulation of the AGE-RAGE and Nrf2 pathways, which may contribute to its observed protective effects against DR.

### 2.8. EJPs Regulate the Expression of VEGF, IL-6, TNF-α, IL-1β, MDA, GSH, CAT, and SOD to Improve Retinal Damage

**Figure 7:**
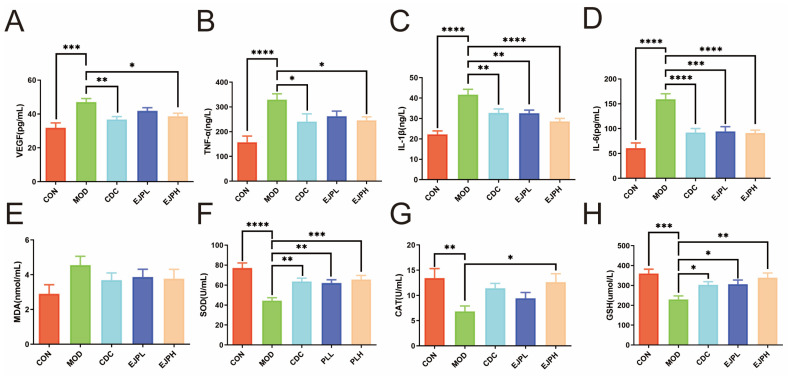
The effects of EJPs on inflammation and oxidative stress in rat serum and retinal tissue. (**A**–**H**) Comparison of serum VEGF, MOD, CAT, SOD, GSH, and retinal tissue IL-6, TNF-α and IL-1β levels in each group of rats. Mean ± SD. * *p* < 0.05, ** *p* < 0.01, *** *p* < 0.001, **** *p* < 0.0001.

Serum VEGF levels were significantly elevated in diabetic rats in the MOD group, consistent with the pathological angiogenesis characteristic of diabetic retinopathy (Figure 7A). EJP intervention suppressed this VEGF overexpression in a dose-dependent manner, suggesting its potential to inhibit retinal neovascularization.

The DR model exhibited a robust inflammatory response, as evidenced by significantly elevated serum levels of TNF-α, IL-6, and IL-1β in the MOD group (Figure 7B–D). Treatment with CDC, EJPL, or EJPH effectively reduced the levels of these pro-inflammatory cytokines, demonstrating the anti-inflammatory properties of EJPs. Diabetic rats displayed profound systemic oxidative stress, characterized by diminished antioxidant defenses (reduced activities of SOD, CAT, and GSH) and enhanced lipid peroxidation (elevated MDA levels) (Figure 7E–H). EJP administration counteracted these abnormalities, indicating the capacity to restore redox homeostasis and alleviate oxidative damage in DR.

## 3. Discussion

Our integrated study demonstrates that the traditional Chinese medicine formula known as EJPs significantly alleviates diabetic retinal injury in rats. The protective effects are manifested as improved retinal structure and function, preservation of the blood–retinal barrier (BRB), and reduction in vascular leakage. Mechanistically, our data point to the coordinated modulation of the AGE-RAGE-NF-κB/Nrf2 axis as a central mechanism underlying EJP efficacy.

The integrity of the BRB is fundamental to retinal homeostasis, and its breakdown is a hallmark of DR, leading to vascular leakage, edema, and neuronal dysfunction [22]. Our study provides multifaceted evidence that EJP treatment effectively preserves BRB function in diabetic rats. First, EJPs significantly mitigated the pathological increase in vascular permeability, as demonstrated by the reduction in sodium fluorescein and Evans blue dye leakage. This functional improvement was paralleled by a marked decrease in serum VEGF levels. VEGF is a well-established master regulator of pathological vascular permeability and angiogenesis in DR, and its overexpression directly compromises BRB integrity by downregulating tight junction proteins [1,23]. Therefore, the suppression of VEGF by EJPs represents a critical mechanism for reducing the destructive force on the retinal vasculature. Second, beyond countering VEGF, EJPs actively fortified the BRB structure itself. We observed a significant upregulation in the expression of key tight junction proteins, including ZO-1, occludin, and claudin-5, in the retinal vasculature of EJP-treated animals. These proteins are essential for forming and maintaining the paracellular seal of endothelial cells, and their loss is directly linked to BRB failure in diabetes [24]. The coordinated action of EJPs—simultaneously dampening the VEGF-driven disruptive signal and enhancing the structural components of the barrier—underscores its multi-targeted efficacy in stabilizing the neurovascular unit during diabetic insult.

Beyond its protective effects on the BRB, our findings elucidate that EJPs intercept diabetic retinal damage at a key upstream pathogenic node: the AGE-RAGE-NF-κB inflammatory axis. Hyperglycemia-driven accumulation of AGEs and their interaction with RAGE initiate a cascade of detrimental events central to DR progression [25]. Consistent with this paradigm, we observed a significant increase in AGE deposition, along with elevated RAGE and NF-κB protein levels, in the retinas of diabetic rats, which was accompanied by a surge in the pro-inflammatory cytokines TNF-α, IL-6, and IL-1β. Critically, EJP treatment disrupted this cascade at multiple levels: it reduced retinal AGE accumulation, downregulated RAGE expression, suppressed NF-κB activation, and consequently diminished the production of downstream inflammatory mediators. This multi-point suppression is significant because the AGE-RAGE interaction is known to activate NF-κB, a master transcriptional regulator of inflammation [26]. The ensuing NF-κB-driven synthesis of cytokines like TNF-α and IL-1β not only sustains a chronic inflammatory milieu but also directly contributes to BRB dysfunction and vascular leakage—thereby bridging the mechanistic action of EJPs to the improved vascular phenotype [27]. By concurrently targeting the AGEs, RAGE, and NF-κB, EJPs demonstrate a comprehensive strategy to quell a primary source of inflammatory insult in the diabetic retina.

In parallel to suppressing inflammation, EJPs robustly augmented the endogenous antioxidant defense system, which is typically compromised in diabetes. The transcription factor Nrf2 serves as the primary regulator of cellular redox homeostasis [28]. Under diabetic conditions, sustained oxidative stress and inflammatory signaling can lead to Nrf2 pathway dysregulation, impairing its nuclear translocation and subsequent transactivation of antioxidant genes [29]. This failure manifests as a depletion of crucial antioxidant enzymes, such as SOD, CAT, and GSH, and an accumulation of lipid peroxidation products like MDA—a profile we confirmed in the retinas of untreated diabetic rats [30]. Treatment with EJPs effectively reversed this imbalance, significantly upregulating the expression and activity of Nrf2 and its key downstream effector HO-1, while restoring the levels of SOD, CAT, and GSH and reducing MDA. This indicates that EJPs actively reinstate the Nrf2-mediated antioxidant program. The therapeutic significance of this action extends beyond mere redox correction. A critical pathogenic feature in DR is the crosstalk between oxidative stress and inflammation, forming a self-perpetuating vicious cycle [31]. Notably, NF-κB and Nrf2 pathways are known to reciprocally inhibit each other; NF-κB activation can suppress Nrf2 activity, while a deficient Nrf2 response exacerbates oxidative stress, further fueling NF-κB-driven inflammation [25,32].

This study is subject to certain limitations. Although network pharmacology predicted that EJPs might interact with multiple targets central to key signaling pathways, such as AKT1, MAPK1, and SRC, our experimental work provided direct validation mainly for the modulation of the AGE-RAGE-NF-κB pathway. The involvement of other predicted targets is yet to be functionally confirmed. Furthermore, the active chemical constituents within the complex mixture of EJPs that drive its therapeutic effects are yet to be precisely identified. To address these gaps, subsequent research will pursue two main directions: first, to functionally dissect the roles of prioritized targets in the protective mechanisms; second, to characterize and quantify the key active components, thereby providing a scientific foundation for the standardization of this herbal preparation.

In conclusion, EJPs mitigate diabetic retinopathy in rats by attenuating oxidative stress, inflammation, and vascular leakage, likely through the coordinated regulation of the AGE-RAGE-NF-κB/Nrf2 axis. This work provides a pharmacological foundation for the traditional use of EJPs and identifies promising directions for further mechanistic investigation and potential standardization.

## 4. Materials and Methods

### 4.1. Main Reagents and Instruments

Chromatography-grade methanol, acetonitrile, formic acid, and acetic acid were obtained from Fisher Scientific (Waltham, MA, USA). Mass spectrometry-grade water, 2-propanol, and 2-chloro-L-phenylalanine were purchased from Merck (Rahway, NJ, USA). Streptozotocin (STZ) and Evans blue were acquired from Sigma-Aldrich (St. Louis, MO, USA). Sodium citrate buffer and sodium fluorescein were sourced from Beijing Solarbio Science & Technology Co., Ltd. (Beijing, China). Tropicamide phenylephrine eye drops were procured from Santen Pharmaceutical Co., Ltd. (Xiamen, China). FAS eye fixative and glutaraldehyde fixative (2.5% in phosphate buffer) were obtained from Servicebio (Wuhan, China). Retinal imaging was performed using the Spectralis HRA + OCT system (Heidelberg Engineering GmbH, Heidelberg, Germany).

### 4.2. Extract of EJP Experimental Medicine

The processed rhizomes of *Polygonatum sibiricum* and the dried fruits of *Lycium barbarum* L. (Lycii Fructus) were purchased from Yunnan Kanglv Agricultural Technology Co., Ltd. (Kunming, China). Their botanical origins were authenticated by a Senior Engineer at the Yunnan University of Chinese Medicine (Voucher specimen numbers: 20230501001 and 20230501002, respectively). The two medicinal materials were ground into a coarse powder (40 mesh) and mixed in a 1:1 (*w*/*w*) ratio. The mixed powder was subjected to water extraction. Briefly, 1000 g of the powder was decocted twice with 10 volumes (*w*/*v*) of distilled water for 2 h per extraction under reflux at 100 °C. The combined aqueous extracts were filtered, concentrated under reduced pressure at 60 °C, and then lyophilized to obtain the dry EJP powder. The extraction yield was approximately 22.5% (*w*/*w*). The entire extraction process was strictly standardized.

### 4.3. EJP Component Characterization

Sample preparation involved adding 300 µL of extraction solvent (methanol:acetonitrile, 1:1, *v*/*v*, spiked with 0.02 mg/mL L-2-chlorophenylalanine (Rahway, NJ, USA) as an internal standard) to a 100 µL aliquot in a 1.5 mL tube. Following extraction, metabolomic analysis was conducted on a UHPLC system coupled to a Q Exactive Orbitrap mass spectrometer (Thermo Scientific, Waltham, MA, USA).

Chromatographic separation was performed on an ACQUITY UPLC BEH C18 column (100 mm × 2.1 mm, 1.7 µm, Waters, Milford, MA, USA) maintained at 40 °C. The mobile phase consisted of solvent A (water with 0.1% formic acid and 2% acetonitrile) and solvent B (acetonitrile with 0.1% formic acid), delivered at a flow rate of 0.3 mL/min. The gradient elution program was as follows: 5–20% B (0–2 min), 20–35% B (2–5 min), 35–65% B (5–10 min), 65–95% B (10–15 min), and 95–5% B (15–17 min), with a 3 µL injection volume.

Mass spectrometric detection was performed in both positive and negative electrospray ionization (ESI) modes using a data-dependent acquisition (DDA) strategy. Key operating parameters were as follows: the spray voltage was set to 3.5 kV (positive) and −3.0 kV (negative); the heater and capillary temperatures were 450 °C and 320 °C, respectively. Full MS scans were acquired at a resolution of 70,000 (*m*/*z* 200) over a mass range of *m*/*z* 70–1050. Data-dependent MS/MS scans were triggered for the top 10 most intense ions, with a resolution of 17,500 and stepped normalized collision energies of 20%, 40%, and 60%. A summary of all parameters is provided in Table S2.

Data were processed using Progenesis QI v3.0 (Waters Corporation, Milford, MA, USA). All metabolite identifications are preliminary annotations based on matching the acquired MS and MS/MS spectra against the MJBIOTCM and an in-house traditional Chinese medicine database, with a mass tolerance of <10 ppm. These annotations have not been validated using authentic chemical standards.

### 4.4. Screening of EJP Chemical Component Targets and Disease Targets

The identified chemical components were input into the SwissADME database. A preliminary screening for active ingredients with potentially good oral bioavailability was conducted by setting the gastrointestinal absorption parameter to “High”. Subsequently, these compounds were evaluated according to five drug-likeness rules (Lipinski, Ghose, Veber, Egan, Muegge). Compounds that received a “Yes” designation in two or more of these rules were considered qualified active compounds. The Swiss Target Prediction tool was then used to match these screened active ingredients with their corresponding potential targets. The term “Diabetic retinopathy” was used to search for related target genes in the GeneCards and OMIM databases. The retrieved targets were pooled, and duplicate entries were removed. The Venny 2.1.0 tool was subsequently employed to identify the common targets shared between the active ingredients and diabetic retinopathy for in-depth analysis. Following this, the drug–component–disease relationships and the intersection targets were organized into type and network files. These files were imported into Cytoscape 3.7.2 to construct a “Component–DiseaseTarget” network figure. The topological properties of this network were studied. Nodes with values of Degree, Betweenness Centrality, and Closeness Centrality all greater than their respective network averages were screened. Among these, the nodes with the highest Degree values were identified as the main active components of EJPs for treating DR [33].

### 4.5. Construction of Protein–Protein Interaction (PPI) Network

Using the STRING database, the intersection genes were first imported into the STRING version 11.5 platform, with the species set to “*Homo sapiens*”. A confidence score threshold of ≥0.9 was applied, and isolated nodes were removed to obtain a visualizable protein–protein interaction relationship map. The connections between the targets were then detailed statistically. Finally, the data were saved and backed up as a TSV format file, which was subsequently imported into Cytoscape 3.7.2 software for visualization of the results.

### 4.6. Gene Ontology (GO) and Kyoto Encyclopedia of Genes and Genomes (KEGG) Enrichment Analyses

Functional enrichment analysis was performed to interpret the common targets. Specifically, Gene Ontology (GO) and KEGG analyses were carried out using the ClusterProfiler package (v3.6.1) to annotate biological attributes and pathways, respectively. The GO analysis interrogates three domains: biological process (BP), molecular function (MF), and cellular component (CC). Terms with a *p*-value < 0.05 were considered significantly enriched.

### 4.7. Molecular Docking

The two-dimensional structures of the candidate compounds (2,4,5,6-Phenanthrenetetrol, 2-Methoxybenzene-1,4-diol, (E)-3-Coumaric acid, 5-Hydroxyindoleacetic Acid, and Paprazine) were retrieved from the PubChem database. Each structure was subjected to geometry optimization via energy minimization using the MMFF94 force field in Chem3D 23.1.1, with a gradient convergence criterion of 0.001 kcal/mol. The energy-minimized structures were subsequently converted into three-dimensional models and saved in PDBQT format using AutoDock Tools 1.5.7.

The three-dimensional crystal structures of the target proteins—HSP90AA1 (PDB: 1UY6), SRC (2SRC), AKT1 (3CQW), PIK3CA (4JPS), and MAPK1 (4QTA)—were obtained from the RCSB Protein Data Bank. Using PyMOL 3.2, all water molecules, co-crystallized ligands, and any heteroatoms (e.g., non-standard residues or ions not involved in catalysis or structural integrity) were removed from each protein structure. Polar hydrogen atoms were added, and Kollman partial atomic charges were assigned. The prepared protein structures were then converted to PDBQT format using AutoDock Tools.

The binding site for docking was defined by centering a grid box on the centroid of the native co-crystallized ligand (or, for structures without a bound ligand, on the centroid of key active site residues as reported in the literature). The grid box dimensions were set to 30 × 30 × 30, with a grid-point spacing of 0.375, ensuring comprehensive sampling of the binding pocket. Molecular docking simulations were performed using AutoDock Vina 1.1.2. The exhaustiveness of the global search was set to 50. For each ligand-protein pair, 20 independent docking runs were conducted. The conformation with the most favorable (i.e., lowest) calculated binding affinity (kcal/mol) was selected for subsequent interaction analysis. The optimal docking poses were visualized using PyMOL 3.2 to elucidate potential molecular interactions, such as hydrogen bonds and hydrophobic contacts.

### 4.8. Experimental Animals and Model

A total of 35 male SPF-grade brown Norway (BN) rats, aged 6 weeks and weighing 150 ± 20 g, were purchased from SPF Biotechnology Co., Ltd. (Beijing, China) (animal production license number: SCXK (Beijing) 2022-0004). The rats were housed in the SPF animal laboratory of the Animal Experiment Center of Yunnan University of Chinese Medicine. The housing conditions were maintained at a room temperature of 20–25 °C, relative humidity of 45–65%, with a 12 h/12 h light-dark cycle and adequate ventilation. All experimental procedures and protocols were approved by the Animal Ethics Committee of Yunnan University of Chinese Medicine (Ethical approval document number: R-062023057).

STZ can selectively destroy pancreatic β-cells and was used to induce diabetes in rats. A freshly prepared STZ solution (30 mg/kg) was administered via intraperitoneal injection for four consecutive days. Fasting blood glucose levels were measured on the 7th day after the last injection. Rats with blood glucose levels consistently ≥16.7 mmol/L were considered diabetic and included in the subsequent study. Following confirmation of successful diabetes induction, the diabetic rats were randomly divided into the following 5 groups (*n* = 7 per group): model group (MOD, received physiological saline), positive control group (CDC, received calcium dobesilate at 135 mg/kg, Beijing, China), low-dose EJPL group (EJPL, 1.5 g/kg), and high-dose EJPL group (EJPH, 6 g/kg). An additional group of age-matched normal rats served as the non-diabetic control (CON, saline). Drug administration commenced on the day following model confirmation (day 13 after the first STZ injection). All treatments were administered orally by gavage once daily in the morning for 10 consecutive weeks. At the end of the 10-week treatment period, blood was collected from the abdominal aorta, and retinal tissues were harvested for subsequent analysis.

The calcium dobesilate was selected as the positive control drug based on its well-established efficacy in diabetic microangiopathy, particularly retinopathy. Its therapeutic actions are primarily attributed to reducing capillary permeability and abnormal blood viscosity. At the molecular level, calcium dobesilate exerts antioxidant effects, inhibits the formation of AGEs, and modulates pathways involving VEGF and nitric oxide (NO), thereby stabilizing the retinal microvasculature [24,34].

### 4.9. Fluorescein Fundus Angiography (FFA) Test

FFA was performed using the Spectralis HRA + OCT system [35]. After anesthesia in rats, 1% tropicamide eye drops (Santen Pharmaceutical Co., Ltd., Xiamen, China) were used to protect the pupils, followed by intraperitoneal injection of 0.1 mL of 10% fluorescein sodium (Solarbio, Beijing, China). The HRA module was used to continuously scan the retina to detect fluorescein sodium leakage in diabetic rats. ImageJ 1.54 software was used to analyze the fluorescence intensity of each group of photos and perform statistical analysis and inter-group comparison (GraphPad Prism v8.0).

### 4.10. Optical Coherence Tomography (OCT) Testing

After anesthesia in rats, 1% tropicamide eye drops were used to dilate the pupils, followed by observation of the changes in retinal structure using the Spectralis HRA + OCT system. Finally, the retinal thickness in OCT images was measured using ImageJ 1.54 software to evaluate the pathological changes in the retina [36].

### 4.11. Determination of EB Penetration

After the last administration, 3 rats were randomly selected from each group for EB permeability testing [37]. Rats were intraperitoneally injected with 2% Evans blue (45 mg/kg, Macklin Biochemical Co., Ltd., Shanghai, China), and after 2 h of circulation, the left ventricle was washed with physiological saline to eliminate circulating EB in the rat’s body. Subsequently, the eyeball was removed, and the retinal tissue was separated under a dissecting microscope (Model ST60, Sunny Optical, Ningbo, China). It was then placed in a 55 °C vacuum drying oven (BPG-9420A, Shanghai Yiheng Scientific Instrument Co., Ltd., Shanghai, China) and dried for 5 h before weighing. The retinal tissue was placed in a 70 °C water bath and 200 μL of formamide (Sinopharm Chemical Reagent Co., Ltd., Shanghai, China) was added to extract EB for 18 h. The extract was centrifuged at 12,000× *g* for 30 min (Model 5428, Eppendorf AG, Hamburg, Germany), and the absorbance of the supernatant at 620 nm was measured using an enzyme-linked immunosorbent assay (ELISA) reader (Epoch HTS2, Agilent Technologies, Inc., Santa Clara, CA, USA). The EB content was calculated through a standard curve (GraphPad Prism v8.0) and compared with the weight of dried retinal tissue to calculate the EB leakage rate.

### 4.12. Hematoxylin and Eosin (H&E) Staining

The rat eyeballs were fixed overnight in FAS eye fixative (Servicebio, Wuhan, China) and then embedded in paraffin and cut into 3 μm continuous sections. The sections were stained with H&E, and the pathological changes of the retina were observed under an upright optical microscope (Nikon Eclipse E100, Nikon Corporation, Tokyo, Japan) to evaluate the degree of diabetic retinopathy. The method for detecting retinal thickness is specified in Section 4.11, and relevant quantitative comparisons were performed using GraphPad Prism v8.0.

### 4.13. Transmission Electron Microscopy Analysis

After euthanizing the rats, the eyeballs were quickly removed and fixed in 2.5% glutaraldehyde (Servicebio, Wuhan, China) for 2–4 h. The retina was then dissected, cut it into small pieces of 1 mm^3^, and fixed in 1% osmium tetroxide (Ted Pella Inc., Redding, CA, USA) in 0.1 M phosphate buffer solution for 2 h. Following dehydration treatment, the tissue was embedded in SPI Pon 812 epoxy resin (SPI Supplies, West Chester, PA, USA) and polymerized at 60 °C for more than 48 h. Ultra-thin sections of 60–80 nm were cut using a Leica UC7 microtome (Leica Microsystems, Wetzlar, Germany), stained with 2% uranyl acetate (Servicebio, Wuhan, China) for 8 h, and finally, retinal ultrastructure changes were observed under a transmission electron microscope (Hitachi HT7800/HT7700, Hitachi High-Technologies Corporation, Tokyo, Japan).

### 4.14. Western Blot Analysis

Retinal tissues stored at −80 °C were homogenized in lysis buffer (prepared in-house) to extract total protein. Protein samples were denatured by boiling at 100 °C for 5 min and cooled to room temperature prior to loading. The denatured proteins were separated by SDS-PAGE and subsequently transferred onto a PVDF membrane (MilliporeSigma, Burlington, MA, USA). The membrane was then blocked with 5% non-fat milk (Solarbio, Beijing, China) for 2 h at room temperature on a shaker. Following blocking, the membrane was incubated overnight at 4 °C with the following primary antibodies: anti-ZO-1 (1:10,000, Cat# 21773-1-AP, Proteintech, San Diego, CA, USA), anti-occludin (1:5000, Cat# 13409-1-AP, Proteintech), anti-claudin-5 (1:10,000, Cat# 29767-1-AP, Proteintech), anti-NF-κB p65 (1:5000, Cat# 10745-1-AP, Proteintech), anti-RAGE (1:2000, Cat# 16346-1-AP, Proteintech), and anti-β-actin (1:5000, Cat# bs-0061R, BIOSS, Woburn, MA, USA). After washing with TBST [Tris-buffered saline with 0.1% Tween-20], the membrane was incubated with an HRP-conjugated goat anti-rabbit IgG secondary antibody (1:5000, Cat# SA00001-2, Proteintech) for 1 h at room temperature. Protein bands were visualized using an enhanced chemiluminescence substrate (GLPBIO, San Diego, CA, USA). The optical density of each target protein band was quantified using ImageJ 1.54 software and normalized to that of β-actin to determine the relative expression level.

### 4.15. IHC Staining

Paraffin-embedded retinal sections were routinely deparaffinized and rehydrated. After three 5 min washes with phosphate-buffered saline (PBS; Solarbio, Beijing, China), endogenous peroxidase activity was quenched by incubation with 3% hydrogen peroxide (prepared in-house) for 30 min. The sections were then blocked with 3% bovine serum albumin (BSA; Sigma-Aldrich, St. Louis, MO, USA) for 30 min at room temperature. Subsequently, the sections were incubated with a rabbit anti-ZO-1 primary antibody (1:2000, Cat# 21773-1-AP, Proteintech, Proteintech, San Diego, CA, USA) overnight at 4 °C. After washing with PBS, the sections were incubated with an HRP-conjugated goat anti-rabbit IgG secondary antibody (1:200, Cat# GB23303, Servicebio, Wuhan, China) for 1 h at room temperature. Color development was performed using a 3,3′-diaminobenzidine substrate kit (ZSGB-Bio, Beijing, China), and the reaction was terminated with distilled water after approximately 2–5 min. Finally, the sections were counterstained with hematoxylin (Solarbio, Beijing, China), dehydrated, cleared in xylene (Sinopharm Chemical Reagent, Shanghai, China), and mounted with neutral balsam (Solarbio, Beijing, China). For quantitative analysis, three non-overlapping fields per section were randomly selected under a light microscope (Nikon Eclipse E100, Nikon Corporation, Tokyo, Japan). The positive staining (brown-yellow) was analyzed using ImageJ 1.54 software. The expression level of ZO-1 was evaluated by calculating the average optical density (AOD) using the formula AOD = integrated optical density (IOD)/total area.

### 4.16. Immunofluorescence

Retinal sections were processed for immunofluorescence. After deparaffinization, rehydration, and antigen retrieval, sections were blocked with 3% BSA (Solarbio, Beijing, China) for 1 h at room temperature. The sections were then incubated with a rabbit anti-AGE primary antibody (1:100, Cat# bs-1158R, BIOSS, Beijing, China) overnight at 4 °C in a humidified chamber. After three 5 min washes with PBS (Solarbio, Beijing, China), the sections were incubated with a fluorescently labeled secondary antibody (1:200, Cat# GB22303, Servicebio, Wuhan, China) for 50 min at room temperature in the dark. Following another series of PBS washes, cell nuclei were counterstained with DAPI (1 µg/mL; Servicebio, Wuhan, China) for 10 min. To reduce tissue autofluorescence, sections were treated with an autofluorescence quenching reagent() for 5 min and rinsed under running tap water for 10 min. Finally, sections were mounted with an anti-fade mounting medium (Servicebio, Wuhan, China).

Fluorescent images were acquired using a BX51 inverted fluorescence microscope (OLYMPUS, Tokyo, Japan). For each retinal section, three non-overlapping fields were captured at 200× magnification. The Mean Fluorescence Intensity (MFI) of AGE immunostaining was quantified using ImageJ 1.54 software.

### 4.17. RT-qPCR

Utilizing a total RNA extraction kit (Tiangen Biotech, Beijing, China) according to the manufacturer’s protocol, the concentration and purity of the extracted RNA were assessed by measuring the absorbance at 260 nm and 280 nm using a NanoDrop spectrophotometer (Thermo Fisher Scientific, Waltham, MA, USA). RNA samples with an A260/A280 ratio between 1.8 and 2.0 were considered suitable for subsequent experiments. First-strand complementary DNA was synthesized from 1 µg of total RNA using the Hifair^®^ III 1st Strand cDNA Synthesis SuperMix (YEASEN, Shanghai, China) in a 20 µL reaction volume, following the manufacturer’s instructions. The reverse transcription conditions were as follows: 42 °C for 2 min. Quantitative PCR was performed on a QuantStudio™ 1 Plus Real-Time PCR System (Thermo Scientific, Waltham, MA, USA) using Hieff UNICON^®^ Universal Blue SYBR Green Master Mix (YEASEN, Shanghai, China). The 20 µL PCR reaction mixture contained 10 µL of SYBR Green Master Mix, 0.8 µL of each forward and reverse primer (10 µM), 2 µL of cDNA template, and 6.4 µL of nuclease-free water. The primer sequences used in this study are listed below (all primers were synthesized by Sangon Biotech, Shanghai, China):

*Nrf2*: Forward: 5′-GCCTTCCTCTGCTGCCATTAGTC-3′, Reverse: 5′-TGCCTTCAGTGTGCTTCTGGTTG-3′.

*HO-1*: Forward: 5′-AGGGTCAGGTGTCCAGGGAAAG-3′, Reverse: 5′-TCTGCTTGTTTCGCTCTATCTCCCTC-3‘.

*RAGE*: Forward: 5′-CCCAATGGTTCACTCCTCCTTCC-3′, Reverse: 5′-ACTTGGTAGACTCGGACTCGGTAG-3′.

*β-actin*: Forward: 5′-GTGACGTTGACATCCGTAAAGA-3′, Reverse: 5′-GCCGGACTCATCGTACTCC-3′.

The relative mRNA expression levels of target genes were normalized to the endogenous reference gene *β-actin* and calculated using the 2^−ΔΔCt^ method. All reactions were performed in triplicate for each sample.

### 4.18. Biological Kit Determination

Serum and retinal tissues were collected and stored at −80 °C until analysis. After thawing, the levels of inflammatory cytokines (TNF-α, IL-6, and IL-1β) in retinal tissue homogenates and VEGF in serum were measured using specific enzyme-linked immunosorbent assay (ELISA) kits according to the manufacturers’ instructions. The commercial ELISA kits used were as follows: VEGF (Cat# MM-0807R2), TNF-α (Cat# MM-0180R1), IL-6 (Cat# MM-0190R1), and IL-1β (Cat# MM-0047R1), all from Jiangsu Meimian Industrial Co., Ltd. (Zhangjiagang, China).

The levels of oxidative stress markers, including malondialdehyde (MDA), superoxide dismutase (SOD), catalase (CAT), and glutathione (GSH), in serum were determined using corresponding colorimetric assay kits (MDA, Cat# A003-1-2; SOD, Cat# A001-3-2; CAT, Cat# A007-1-1; GSH, Cat# A006-2-1) purchased from the Nanjing Jiancheng Bioengineering Institute (Nanjing, China).

### 4.19. Statistical Analysis

Data are presented as the mean ± standard deviation (SD). Statistical analyses were performed using GraphPad Prism software (version 8.0). For comparisons between the MOD group and all other groups, one-way analysis of variance (ANOVA) was used, followed by Dunnett’s post hoc test. A *p*-value of less than 0.05 was considered statistically significant.

## 5. Conclusions

In conclusion, the role of EJPs in improving retinal damage in diabetes has been verified in many aspects. EJPs improve retinal damage through various mechanisms, such as reducing vascular leakage, protecting retinal structure, increasing the number of RGCs, enhancing tight junction protein expression, and reducing VEGF levels. These findings provide a strong scientific basis for EJPs as a therapeutic strategy for diabetic retinopathy. Future research will further reveal the deeper mechanisms of action, providing a more solid scientific basis for clinical applications.

## Data Availability

The original contributions presented in this study are included in the article/Supplementary Materials. Further inquiries can be directed to the corresponding author.

## Supplementary Materials

The following supporting information can be downloaded at: https://www.mdpi.com/article/10.3390/ph19060940/s1. Table S1: The 408 components were found in EJP. Table S2. Key operating parameters of the Q Exactive Orbitrap mass spectrometer.

## References

[1] Antonetti D.A., Silva P.S., Stitt A.W. (2021). Current understanding of the molecular and cellular pathology of diabetic retinopathy. Nat. Rev. Endocrinol..

[2] Ji L., Tian H., Webster K.A., Li W. (2021). Neurovascular regulation in diabetic retinopathy and emerging therapies. Cell. Mol. Life Sci..

[3] Llorián-Salvador M., Cabeza-Fernández S., Gomez-Sanchez J.A., de la Fuente A.G. (2024). Glial cell alterations in diabetes-induced neurodegeneration. Cell. Mol. Life Sci..

[4] Feng Y., Busch S., Gretz N., Hoffmann S., Hammes H.P. (2012). Crosstalk in the retinal neurovascular unit—Lessons for the diabetic retina. Exp. Clin. Endocrinol. Diabetes.

[5] Jonsson K.B., Frydkjaer-Olsen U., Grauslund J. (2016). Vascular Changes and Neurodegeneration in the Early Stages of Diabetic Retinopathy: Which Comes First?. Ophthalmic Res..

[6] Sha L., Zhao Y., Li S., Wei D., Tao Y., Wang Y. (2024). Insights to Ang/Tie signaling pathway: Another rosy dawn for treating retinal and choroidal vascular diseases. J. Transl. Med..

[7] Yating T., Yongmei X., Panyue Q., Zhengyang Z., Jiankang M., Xingxin Y., Jingping L., Wen G., Jie Y. (2021). Analysis of Medicine Compatibility in Traditional Huangjing Prescriptions. Chin. J. Ethnomed. Ethnopharmacy.

[8] Ren Y., Liang H., Xie M., Zhang M. (2024). Natural plant medications for the treatment of retinal diseases: The blood-retinal barrier as a clue. Phytomedicine.

[9] Zhao P., Zhao C., Li X., Gao Q., Huang L., Xiao P., Gao W. (2018). The genus *Polygonatum*: A review of ethnopharmacology, phytochemistry and pharmacology. J. Ethnopharmacol..

[10] Wang Y., Qin S., Pen G., Chen D., Han C., Miao C., Lu B., Su C., Feng S., Li W. (2017). Original Research: Potential ocular protection and dynamic observation of *Polygonatum sibiricum* polysaccharide against streptozocin-induced diabetic rats’ model. Exp. Biol. Med..

[11] Cheng J., Zhou Z.W., Sheng H.P., He L.J., Fan X.W., He Z.X., Sun T., Zhang X., Zhao R.J., Gu L. (2015). An evidence-based update on the pharmacological activities and possible molecular targets of *Lycium barbarum* polysaccharides. Drug Des. Dev. Ther..

[12] Rjeibi I., Feriani A., Ben Saad A., Ncib S., Sdayria J., Saidi I., Souid S., Hfaiedh N., Allagui M.S. (2017). Phytochemical characterization and bioactivity of *Lycium europaeum*: A focus on antioxidant, antinociceptive, hepatoprotective and nephroprotective effects. Biomed. Pharmacother..

[13] Wang Y., Lan C., Liao X., Chen D., Song W., Zhang Q. (2019). *Polygonatum sibiricum* polysaccharide potentially attenuates diabetic retinal injury in a diabetic rat model. J. Diabetes Investig..

[14] Xing X., Liu F., Xiao J., So K.F. (2016). Neuro-protective Mechanisms of *Lycium barbarum*. Neuromol. Med..

[15] Xiong M., Peng J., Zhou S., Gao Q., Lu J., Ou C., Song H., Peng Q. (2025). *Lycium barbarum* L.: A potential botanical drug for preventing and treating retinal cell apoptosis. Front. Pharmacol..

[16] Song M.K., Roufogalis B.D., Huang T.H. (2012). Reversal of the Caspase-Dependent Apoptotic Cytotoxicity Pathway by Taurine from *Lycium barbarum* (Goji Berry) in Human Retinal Pigment Epithelial Cells: Potential Benefit in Diabetic Retinopathy. Evid.-Based Complement. Altern. Med..

[17] Khalid M., Petroianu G., Adem A. (2022). Advanced Glycation End Products and Diabetes Mellitus: Mechanisms and Perspectives. Biomolecules.

[18] Pal R., Bhadada S.K. (2023). AGEs accumulation with vascular complications, glycemic control and metabolic syndrome: A narrative review. Bone.

[19] Kowluru R.A., Kowluru A., Mishra M., Kumar B. (2015). Oxidative stress and epigenetic modifications in the pathogenesis of diabetic retinopathy. Prog. Retin. Eye Res..

[20] Zhu H., Li B., Huang T., Wang B., Li S., Yu K., Cai L., Ye Y., Chen S., Zhu H. (2025). Update in the molecular mechanism and biomarkers of diabetic retinopathy. Biochim. Biophys. Acta Mol. Basis Dis..

[21] Simó R., Stitt A.W., Gardner T.W. (2018). Neurodegeneration in diabetic retinopathy: Does it really matter?. Diabetologia.

[22] Barber A.J. (2003). A new view of diabetic retinopathy: A neurodegenerative disease of the eye. Prog. Neuropsychopharmacol. Biol. Psychiatry.

[23] Simó R., Sundstrom J.M., Antonetti D.A. (2014). Ocular Anti-VEGF therapy for diabetic retinopathy: The role of VEGF in the pathogenesis of diabetic retinopathy. Diabetes Care.

[24] Leal E.C., Martins J., Voabil P., Liberal J., Chiavaroli C., Bauer J., Cunha-Vaz J., Ambrósio A.F. (2010). Calcium dobesilate inhibits the alterations in tight junction proteins and leukocyte adhesion to retinal endothelial cells induced by diabetes. Diabetes.

[25] Kang Q., Yang C. (2020). Oxidative stress and diabetic retinopathy: Molecular mechanisms, pathogenetic role and therapeutic implications. Redox Biol..

[26] Santiago A.R., Boia R., Aires I.D., Ambrósio A.F., Fernandes R. (2018). Sweet Stress: Coping With Vascular Dysfunction in Diabetic Retinopathy. Front. Physiol..

[27] Aveleira C.A., Lin C.M., Abcouwer S.F., Ambrósio A.F., Antonetti D.A. (2010). TNF-α signals through PKCζ/NF-κB to alter the tight junction complex and increase retinal endothelial cell permeability. Diabetes.

[28] Kensler T.W., Wakabayashi N., Biswal S. (2007). Cell survival responses to environmental stresses via the Keap1-Nrf2-ARE pathway. Annu. Rev. Pharmacol. Toxicol..

[29] Ghosh A., Abdo S., Zhao S., Wu C.H., Shi Y., Lo C.S., Chenier I., Alquier T., Filep J.G., Ingelfinger J.R. (2017). Insulin Inhibits *Nrf2* Gene Expression via Heterogeneous Nuclear Ribonucleoprotein F/K in Diabetic Mice. Endocrinology.

[30] Tan B.L., Norhaizan M.E., Liew W.P., Sulaiman Rahman H. (2018). Antioxidant and Oxidative Stress: A Mutual Interplay in Age-Related Diseases. Front. Pharmacol..

[31] Kowluru R.A. (2023). Cross Talks between Oxidative Stress, Inflammation and Epigenetics in Diabetic Retinopathy. Cells.

[32] Gao W., Guo L., Yang Y., Wang Y., Xia S., Gong H., Zhang B.K., Yan M. (2021). Dissecting the Crosstalk Between *Nrf2* and NF-κB Response Pathways in Drug-Induced Toxicity. Front. Cell Dev. Biol..

[33] He X.D., Li M., Zuo X.D., Ni H.Y., Han Y.X., Hu Y.K., Yu J., Yang X.X. (2025). Kushenol I combats ulcerative colitis via intestinal barrier preservation and gut microbiota optimization. World J. Gastroenterol..

[34] Lameynardie S., Chiavaroli C., Travo P., Garay R.P., Parés-Herbuté N. (2005). Inhibition of choroidal angiogenesis by calcium dobesilate in normal Wistar and diabetic GK rats. Eur. J. Pharmacol..

[35] Zang X., Zhang L., Ma J., Wang A., Ding L., Wang Y., Sun J., Li J., Hang X., Li X. (2025). Mechanistic Insights into Shenzhuo Formula for Diabetic Retinopathy: Integrating UPLC-Q-TOF-MS/MS, Network Pharmacology, Single-Cell RNA Sequencing Data, and Experimental Validation. Drug Des. Dev. Ther..

[36] Zhou P., Xie W., Meng X., Zhai Y., Dong X., Zhang X., Sun G., Sun X. (2019). Notoginsenoside R1 Ameliorates Diabetic Retinopathy through *PINK1*-Dependent Activation of Mitophagy. Cells.

[37] Fang M., Wan W., Li Q., Wan W., Long Y., Liu H., Yang X. (2021). Asiatic acid attenuates diabetic retinopathy through TLR4/MyD88/NF-κB p65 mediated modulation of microglia polarization. Life Sci..

