# Supplementary material for: Ameliorative Effect of Erjing Pills on Retinal Damage in Rats with Diabetic Retinopathy
Source: Pharmaceuticals (Basel). 2026 Jun 15;19(6):940. doi: 10.3390/ph19060940 (PMC13305654; doi:10.3390/ph19060940)
Supplement: Supplementary file 1 [file pharmaceuticals-19-00940-s001.zip › TableS1.pdf]

The 408 components were found in EJP Table S1

NO.	Metabolite	Formula	Retention time	Mode	Adducts	m/z	Mass Error (ppm)
metab_0	Choline	C5H14NO+	0.5631	pos	M+	104.1073	-2.862237894
metab_1	1-Deoxynojirimycin	C6H13NO4	0.5848	pos	M+H-H2O	146.081	-0.808907613
metab_3	Furfural	C5H4O2	0.6029	pos	M+H	97.0288	4.1718025084459
metab_4	3-hydroxy-3-methyl-Glutaric acid	C6H10O5	0.6029	pos	M+H	163.06	-0.870274756
metab_5	D-Glucosamine	C6H13NO5	0.6029	pos	M+H	180.0865	-0.796925354
metab_6	4-aminobenzoic acid	C7H7NO2	0.6215	pos	M+H	138.0549	-0.442540261
metab_7	4-hydroxyproline	C5H9NO3	0.6613	pos	M+H-2H2O	96.0448	3.0245555881243
metab_8	Adenine	C5H5N5	0.6613	pos	M+H	136.0618	0.19935481222764
metab_9	Cyclohexanone	C6H10O	0.6791	pos	M+H	99.0808	4.0195322169169
metab_11	Isonicotinic acid	C6H5NO2	0.6974	pos	M+H	124.0395	1.234774543
metab_12	D-Pyroglutamic acid	C5H7NO3	0.6974	pos	M+H	130.0499	0.56042790664774
metab_13	2-Phenylacetamide	C8H9NO	0.6974	pos	M+H	136.0757	-0.012871122
metab_14	4-Trimethylammoniobutanoic Acid	C7H15NO2	0.6974	pos	M+H	146.1175	-0.424523809
metab_15	Uracil	C4H4N2O2	0.9635	pos	M+H	113.0349	2.7068210612976
metab_16	Tyramine	C8H11NO	2.0497	pos	M+H-H2O	120.081	1.3724823029842
metab_17	6-methyluracil	C5H6N2O2	2.0497	pos	M+H	127.0498	-3.444418838
metab_18	5-Hydroxymethyl-2-Furaldehyde	C6H6O3	2.0931	pos	M+H	127.0391	1.1753030067524
metab_19	2-Methoxyhydroquinone	C7H8O3	2.7595	pos	M+H	141.0547	0.32159721124801
metab_20	Mevalonic acid	C6H12O4	2.7817	pos	M+H-2H2O	113.06	2.0600931383913
metab_21	L-tyrosine	C9H11NO3	2.825	pos	M+H	182.0813	0.69808031590544
metab_22	Indole-3-carboxaldehyde	C9H7NO	2.8469	pos	M+H	146.0601	0.50700907580744
metab_23	5-Hydroxyindoleacetic acid	C10H9NO3	2.8469	pos	M+H	192.0656	0.18228199325236

NO.	Metabolite	Formula	Retention time	Mode	Adducts	m/z	Mass Error (ppm)
metab_24	Arecoline	C8H13NO2	2.9335	pos	M+H	156.102	0.77792536261053
metab_25	Anthranilic acid	C7H7NO2	3.0671	pos	M+H	138.055	0.63302149202506
metab_26	Sorbic acid	C6H8O2	3.1776	pos	M+H-H2O	95.0496	4.2823466261197
metab_27	Piceol	C8H8O2	3.1776	pos	M+H	137.0598	0.75076134619056
metab_28	Serotonin	C10H12N2O	3.1776	pos	M+H	177.1023	0.57461859582341
metab_29	L-Homotyrosine	C10H13NO3	3.2213	pos	M+H	196.0969	0.6483129533773
metab_30	3-Hydroxypicolinic acid	C6H5NO3	3.2656	pos	M+H-H2O	122.0239	1.6530145984198
metab_31	1-(4-methylphenyl)ethanone	C9H10O	3.3315	pos	M+H-H2O	117.0702	2.1682703843391
metab_32	Picolinic acid	C6H5NO2	3.3315	pos	M+H	124.0395	1.9409309470731
metab_33	Indoline	C8H9N	3.3765	pos	M+H	120.0811	2.3410993648985
metab_34	4-allylanisole	C10H12O	3.4214	pos	M+NH4	166.1228	0.88860461212514
metab_35	Scytalone	C10H10O4	3.4886	pos	M+H	195.0654	1.0745034445364
metab_36	2-aminoacetophenone	C8H9NO	3.5755	pos	M+H	136.0758	0.88383020107345
metab_37	4-Hydroxybenzaldehyde	C7H6O2	3.6424	pos	M+H	123.0443	2.0475573736489
metab_38	Norharman	C11H8N2	3.665	pos	M+H	169.0761	0.67511902024646
metab_39	Acetosyringone	C10H12O4	3.71	pos	M+Na	219.0643	7.9886444714099
metab_40	Securinine	C13H15NO2	3.7543	pos	M+NH4	235.1442	0.27218045503827
metab_41	Acetophenone	C8H8O	3.9568	pos	M+H	121.0651	2.3861905357472
metab_42	4-Hydroxycinnamyl aldehyde	C9H8O2	3.9568	pos	M+H	149.0599	1.1355923060544
metab_43	Phenylacetic acid	C8H8O2	3.9787	pos	M+H-H2O	119.0494	2.1461493613722
metab_44	4-Coumaric acid	C9H8O3	3.9787	pos	M+H	165.0548	1.0276792498287
metab_45	Glutamylphenylalanine	C14H18N2O5	4.0232	pos	M+H-2H2O	259.1078	0.23176947541942
metab_46	Isovanillin	C8H8O3	4.0453	pos	M+H	153.0547	0.76074438417877
metab_47	N-methylanthranilic acid	C8H9NO2	4.1341	pos	M+H	152.0707	0.70870924846444
metab_48	Syringic acid	C9H10O5	4.4883	pos	M+H-H2O	181.0496	0.38717999117429

NO.	Metabolite	Formula	Retention time	Mode	Adducts	m/z	Mass Error (ppm)
metab_49	1-phenylpropane-1,2-dione	C9H8O2	4.5107	pos	M+H	149.0598	0.87164971192805
metab_50	Indole-3-acetaldehyde	C10H9NO	4.5557	pos	M+ACN+H	201.1024	0.97368439887098
metab_51	Protopine	C20H19NO5	4.6465	pos	M+H	354.1333	-0.806736157
metab_52	Khellactone	C14H14O5	4.6692	pos	M+H-H2O	245.0809	0.16105883566174
metab_53	O-Cymol	C10H14	4.6911	pos	M+H	135.1169	0.88166594037957
metab_54	Benzyl butyrate	C11H14O2	4.6911	pos	M+H	179.1067	0.45710864669738
metab_55	Loliolide	C11H16O3	4.6911	pos	M+H	197.1173	0.47877990926412
metab_56	Phenylethyl beta-d-glucopyranoside	C14H20O6	4.6911	pos	M+H	285.1307	-9.017462236
metab_57	Indole	C8H7N	4.7365	pos	M+H	118.0654	2.3274378001453
metab_58	2,4,5,6-Tetrahydroxyphenanthrene	C14H10O4	4.8051	pos	M+H	243.0653	0.27064730501536
metab_59	Hymecromone methyl ether	C11H10O3	4.8279	pos	M+H	191.0704	0.49113482651244
metab_60	Iso-gamma-fagarine	C13H11NO3	4.8507	pos	M+H	230.0811	-0.282911576
metab_61	4-Methoxycinnamaldehyde	C10H10O2	4.9183	pos	M+H	163.0754	0.44371084710364
metab_62	Microminutin	C15H12O5	4.9868	pos	M+H	273.0757	-0.091170088
metab_63	3-Coumaric acid	C9H8O3	5.3461	pos	M+H-H2O	147.0441	0.30903714947864
metab_64	N-P-Coumaroyltyramine	C17H17NO3	5.3461	pos	M+H	284.1279	-0.63841796
metab_65	Plastoquinone-1	C13H16O2	5.4124	pos	M+H	205.1223	-0.06629486
metab_66	Senkyunolide G	C12H16O3	5.4809	pos	M+H	209.1172	-0.085555092
metab_67	4-Methylumbelliferone	C10H8O3	5.5715	pos	M+H	177.0546	0.14921067886462
metab_68	Ephedrine	C10H15NO	5.7988	pos	M+H-H2O	148.1121	-0.00410797
metab_69	Arglabin	C15H18O3	5.8674	pos	M+H	247.1328	-0.170431943
metab_70	2-Propenal, 3-(1,3-benzodioxol-5-yl)-	C10H8O3	5.9589	pos	M+H	177.0547	0.20611804919434
metab_71	4-Isopropylbenzaldehyde	C10H12O	6.3931	pos	M+H	149.0961	0.3719130919809
metab_72	Dihydroactinidiolide	C11H16O2	6.4845	pos	M+H	181.1223	-0.110972292

NO.	Metabolite	Formula	Retention time	Mode	Adducts	m/z	Mass Error (ppm)
metab_73	2-buten-1-one, 1-(2,6,6-trimethyl-1,3-cyclohexadien-1-yl)-	C13H18O	6.4845	pos	M+H	191.143	-0.142138846
metab_74	Cinnamic acid	C9H8O2	6.5073	pos	M+H-H2O	131.0492	0.68131290223459
metab_75	Cannabisin D	C36H36N2O8	6.5759	pos	M+H	625.2545	0.16339355745369
metab_76	Aschantin	C22H24O7	6.5987	pos	M+H	401.1593	-0.498636222
metab_77	Parthenicin	C15H18O4	6.6216	pos	M+H	263.1277	-0.498153524
metab_78	5,7-dimethoxycoumarin	C11H10O4	7.878	pos	M+H	207.0652	-0.088857194
metab_79	Myristic acid	C14H28O2	8.0152	pos	M+NH4	246.2426	-0.724399084
metab_80	Fluoren-9-one	C13H8O	8.0608	pos	M+H	181.0648	-0.173451388
metab_81	Alpha-Linolenic Acid	C18H30O2	8.175	pos	M+H	279.2317	-0.739225748
metab_82	Alpha-estradiol	C18H24O2	8.3577	pos	M+H	273.1847	-0.908435919
metab_83	Alpha-pinene oxide	C10H16O	8.5857	pos	M+H-H2O	135.1169	0.25363116559993
metab_84	Ectocarpene	C11H16	8.5857	pos	M+H	149.1325	0.24878763529391
metab_85	Boschnialactone	C9H14O2	8.6306	pos	M+H	155.1066	-0.221072378
metab_86	4-n-Hexylphenol	C12H18O	8.6306	pos	M+H	179.143	-0.076188732
metab_87	Sclareol Glycol	C16H30O2	8.6306	pos	M+Na	277.216	8.8344207279476
metab_88	6-Methyl-7-(3-oxobutyl)-bicyclo[4.1.0]heptan-3-one	C12H18O2	9.6125	pos	M+H	195.138	0.35787973514852
metab_89	Octadecenedioic acid	C18H32O4	10.0924	pos	M+H	313.2371	-0.683626313
metab_90	Phytosphingosine	C18H39NO3	10.7544	pos	M+H	318.3001	-0.593411139
metab_92	Cis-7-hexadecenoic acid	C16H30O2	11.4852	pos	M+Na	277.2161	8.9908289412933
metab_93	4-(2,6,6-Trimethylcyclohexa-1,3-dienyl)but-3-en-2-one	C13H18O	11.6905	pos	M+H	191.143	-0.028881625
metab_94	Pterosin g	C14H18O3	12.1239	pos	M+H	235.1328	-0.387938334
metab_96	Sphinganine	C18H39NO2	12.3522	pos	M+H	302.3052	-0.464989184
metab_97	Gorlic acid	C18H30O2	12.4657	pos	M+H	279.2318	-0.279633686
metab_98	Sedanonic acid lactone	C12H16O2	13.0344	pos	M+H	193.1224	0.37468081573889

NO.	Metabolite	Formula	Retention time	Mode	Adducts	m/z	Mass Error (ppm)
metab_99	Benzylideneacetone	C10H10O	13.6038	pos	M+H-H2O	129.07	0.64260020766714
metab_100	6-Pentadecylsalicylic acid	C22H36O3	13.6724	pos	M+H	349.2736	-0.476662536
metab_101	Arbutin	C12H16O7	13.7864	pos	M+H	273.0966	-0.936402749
metab_102	Tetralin	C10H12	13.8979	pos	M+H	133.1012	0.28551651205752
metab_103	Butyrophenone	C10H12O	13.8979	pos	M+H	149.096	-0.304112252
metab_104	Hexadecanamide	C16H33NO	14.2231	pos	M+H	256.2633	-0.715011856
metab_105	Erucamide	C22H43NO	14.5419	pos	M+H	338.3415	-0.701232903
metab_107	Bis(2-ethylhexyl) phthalate	C24H38O4	15.2085	pos	M+H	391.2841	-0.548790054
metab_111	Phloroglucinol	C6H6O3	16.0032	pos	M+H	127.0391	1.1221539701149
metab_112	4-Hydroxybenzoylcholine	C12H18NO3+	15.437	pos	M+	224.128	-3.217677106
metab_113	5-Ethyl-2-methylpyridine	C8H11N	15.3225	pos	M+H	122.0966	1.2861358014319
metab_115	Adipic acid	C6H10O4	15.2543	pos	M+H	147.0651	-0.374019
metab_118	Protocatechuic aldehyde	C7H6O3	15.2085	pos	M+H-H2O	121.0285	0.96462240326605
metab_119	2,4-dimethylaniline	C8H11N	14.6072	pos	M+NH4	139.123	0.10643088360273
metab_121	(-)-gamma-ionone	C13H20O	14.4499	pos	M+H	193.1587	0.27376264691409
metab_122	Sphingosine	C18H37NO2	14.2907	pos	M+H-H2O	282.279	-0.555350512
metab_123	Palmitoyl ethanolamide	C18H37NO2	14.055	pos	M+H	300.2896	-0.416207802
metab_124	Eucommiol	C9H16O4	13.8979	pos	M+H-2H2O	153.0909	-0.607478823
metab_125	Linolenyl alcohol	C18H32O	13.7864	pos	M+NH4	282.2789	-0.912863764
metab_126	Diisobutylphthalate	C16H22O4	13.4894	pos	M+H	279.1589	-0.561709669
metab_128	Stearidonic acid	C18H28O2	13.4438	pos	M+H	277.2161	-0.230414722
metab_130	Thymoquinone	C10H12O2	12.9892	pos	M+H	165.0911	0.44093725530706
metab_131	9-Hydroxy-10E,12Z-octadecadienoic acid	C18H32O3	12.2837	pos	M+H	297.2423	-0.356235042
metab_132	Gamma-Linolenic acid	C18H30O2	11.9414	pos	M+H	279.2317	-0.483726795
metab_133	Curcumenol	C15H22O2	11.4395	pos	M+H-H2O	217.1587	0.24650446020828

NO.	Metabolite	Formula	Retention time	Mode	Adducts	m/z	Mass Error (ppm)
metab_134	Grandifloric acid	C20H30O3	10.8916	pos	M+H	319.2267	-0.316686914
metab_135	Auraptene	C19H22O3	9.9781	pos	M+Na	321.1459	-0.709956283
metab_136	Neocnidilide	C12H18O2	9.9324	pos	M+H	195.138	0.17080168714442
metab_138	17-Hydroxylinolenic acid	C18H30O3	9.6125	pos	M+H	295.2267	-0.313494909
metab_139	1,3-dicyclohexylurea	C13H24N2O	9.5668	pos	M+H	225.196	-0.403209932
metab_140	Pseudoionone	C13H20O	9.5439	pos	M+H	193.1588	0.52032080784023
metab_141	9-Oxo-10,12-octadecadienoic acid	C18H30O3	8.6306	pos	M+H	295.2266	-0.61256308
metab_142	1-Acetyl-Beta-Carboline	C13H10N2O	8.6306	pos	M+H	211.0865	-0.283889219
metab_143	Carvacrol	C10H14O	8.6306	pos	M+H	151.1117	-0.024882129
metab_144	Cnidilide	C12H18O2	8.5857	pos	M+H	195.138	0.10152097115117
metab_145	3,4-Dimethylstyrene	C10H12	8.5857	pos	M+H	133.1012	0.55278570487032
metab_146	Nortricycloekasantalic acid	C11H16O2	8.4034	pos	M+H	181.1223	-0.127467623
metab_147	Confertifoline	C15H22O2	7.9922	pos	M+H	235.1691	-0.545324975
metab_148	Jasmone	C11H16O	6.9866	pos	M+H	165.1274	0.067935936474536
metab_150	2-methoxybenzoic acid	C8H8O3	6.827	pos	M+H-H2O	135.0442	0.65083611852624
metab_151	Resveratrol	C14H12O3	6.8046	pos	M+H-H2O	211.0753	-0.08476061
metab_152	Acenaphthene	C12H10	6.8046	pos	M+H	155.0855	-0.038171679
metab_153	Valerophenone	C11H14O	6.4845	pos	M+H	163.1117	-0.192467706
metab_154	(S)-(-)-Perillyl alcohol	C10H16O	6.4845	pos	M+H-H2O	135.1169	0.32868629680321
metab_156	Santene	C9H14	6.1875	pos	M+H	123.117	1.4402070784652
metab_157	Gomisin R	C22H24O7	6.1416	pos	M+H	401.1593	-0.506426272
metab_158	D-Cathine	C9H13NO	6.0502	pos	M+H-H2O	134.0965	0.39015167968512
metab_159	Dihydrolycorine	C16H19NO4	5.9131	pos	M+H	290.1386	-0.41620037
metab_160	Qing Hau Sau	C15H22O5	5.8217	pos	M+H	283.1539	-0.439997009
metab_161	Cyclo(phe-leu)	C15H20N2O2	5.7988	pos	M+H	261.1596	-0.506627434
metab_162	7-Amino-4-Methylcoumarin	C10H9NO2	5.7988	pos	M+H	176.0707	0.26414489055276

NO.	Metabolite	Formula	Retention time	Mode	Adducts	m/z	Mass Error (ppm)
metab_163	Trigonelline	C7H8NO2+	5.7988	pos	M+	138.055	-3.853637215
metab_164	4-n-Pentylphenol	C11H16O	5.64	pos	M+H	165.1274	0.3342964418993
metab_165	Salvianolic acid f	C17H14O6	5.6171	pos	M+Na	337.068	-0.952342135
metab_167	Feruloyl tyramine	C18H19NO4	5.2586	pos	M+H	314.1384	-0.899339652
metab_168	Ferulic acid	C10H10O4	5.2586	pos	M+H-H2O	177.0547	0.37336536410445
metab_169	Dihydrokaempferol	C15H12O6	5.2147	pos	M+H	289.0705	-0.61277898
metab_170	Triethyl phosphate	C6H15O4P	5.2147	pos	M+H	183.0781	0.15290731153402
metab_171	4-methoxybenzaldehyde	C8H8O2	5.1698	pos	M+H	137.0598	0.66584877585577
metab_172	3-phenylpropionic acid	C9H10O2	5.1469	pos	M+H	151.0754	0.60977865414573
metab_173	Coumaroyl tyramine	C17H17NO3	5.0554	pos	M+H	284.1281	-0.194563879
metab_174	Ethyl paraben	C9H10O3	4.9183	pos	M+H-H2O	149.0598	0.7080103541412
metab_175	Precocene I	C12H14O2	4.7137	pos	M+H	191.1067	0.46132293187161
metab_176	Carboxymethyl isoferulate	C12H12O6	4.6911	pos	M+H	253.0705	-0.462436132
metab_177	Alpha,4-Dimethylstyrene	C10H12	4.6692	pos	M+H	133.1013	1.2215661285131
metab_178	3-n-Butylphathlide	C12H14O2	4.5332	pos	M+H	191.1068	0.72999623079574
metab_179	Cathinone	C9H11NO	4.4883	pos	M+H	150.0915	0.73906121927271
metab_180	Octopamine (p-Hydroxyphenylethanolamine)	C8H11NO2	4.4666	pos	M+H	154.0863	0.43736976017669
metab_181	Fraxidin	C11H10O5	4.4442	pos	M+H	223.0601	0.073291948891452
metab_182	Herniarin	C10H8O3	4.4442	pos	M+H	177.0548	0.74260703433431
metab_183	N-Malonyltryptophan	C14H14N2O5	4.4218	pos	M+H	291.0974	-0.487484883
metab_184	Sinapyl Alcohol	C11H14O4	4.3543	pos	M+H	211.0966	0.4888442036282
metab_185	2-Methylbenzoic acid	C8H8O2	4.3543	pos	M+H	137.0598	0.77262830516251
metab_186	Scopoletin	C10H8O4	4.3098	pos	M+H	193.0496	0.47176014795626
metab_187	Noradrenaline	C8H11NO3	4.3098	pos	M+H-2H2O	134.0602	0.68932531809393
metab_188	Sarracenin	C11H14O5	4.2221	pos	M+H	227.0912	-0.708070388

NO.	Metabolite	Formula	Retention time	Mode	Adducts	m/z	Mass Error (ppm)
metab_189	3-(2-Hydroxy-4-methoxy-phenyl)-acrylic acid	C10H10O4	4.2221	pos	M+H	195.0652	-0.170021563
metab_190	Tryptophol	C10H11NO	4.1997	pos	M+H	162.0913	-0.089443992
metab_191	2-oxindole	C8H7NO	4.1341	pos	M+H	134.0602	1.2091031522347
metab_192	6-methylcoumarin	C10H8O2	4.0453	pos	M+H	161.0598	0.8689472613377
metab_194	Coumarin	C9H6O2	3.9787	pos	M+H	147.0442	0.94074605479656
metab_195	2-Phenylethyl formate	C9H10O2	3.9118	pos	M+H	151.0755	0.96920381843411
metab_196	Dopamine	C8H11NO2	3.8896	pos	M+H	154.0864	0.80758477653831
metab_197	4-(4-hydroxyphenyl)-2-butanone	C10H12O2	3.7767	pos	M+CH3OH+H	197.1174	1.3453626124944
metab_198	Hydroxytyrosol	C8H10O3	3.7767	pos	M+H	155.0704	0.51726716934143
metab_199	Grasshopper ketone	C13H20O3	3.71	pos	M+H-H2O	207.138	0.38980806572555
metab_200	Xanthoxylin	C10H12O4	3.71	pos	M+H	197.081	1.0783162630952
metab_201	4-Methoxycinnamic acid	C10H10O3	3.665	pos	M+H	179.0704	0.85404736675168
metab_202	Oxyhydrastinine	C11H11NO3	3.5979	pos	M+H	206.0813	0.75369476268832
metab_203	Indole-3-carboxylic acid	C9H7NO2	3.4214	pos	M+H	162.0551	0.96362513028184
metab_204	2-Hydroxycinnamic acid	C9H8O3	3.2656	pos	M+H	165.0548	0.86654734845308
metab_205	Cyclo-(Leu-Thr)	C10H18N2O3	3.2437	pos	M+H-H2O	197.1286	0.45328159672024
metab_206	6-hydroxynicotinic acid	C6H5NO3	3.2437	pos	M+H	140.0343	0.52219029877789
metab_207	5-hydroxyindole	C8H7NO	3.1552	pos	M+H	134.0602	0.99151954977285
metab_208	4-Hydroxycinnamamide	C9H9NO2	2.8469	pos	M+H	164.0707	0.64851873638483
metab_209	Salicylaldehyde	C7H6O2	2.7595	pos	M+H	123.0443	1.6816781426475
metab_210	3-Indoleacetonitrile	C10H8N2	2.4944	pos	M+H	157.0761	0.67355728983023
metab_211	Pyrogallol	C6H6O3	2.0714	pos	M+H-H2O	109.0287	2.5164851585772
metab_212	4-Methyl-5-thiazoleethanol	C6H9NOS	1.6128	pos	M+H	144.0478	0.56980955321271
metab_214	L-phenylalanine	C9H11NO2	0.9635	pos	M+H	166.0863	0.4387922657922
metab_216	Anabasine	C10H14N2	0.9426	pos	M+H	163.123	0.17555932981881

NO.	Metabolite	Formula	Retention time	Mode	Adducts	m/z	Mass Error (ppm)
metab_217	(-)-Nicotine	C10H14N2	0.6974	pos	M+H	163.1229	-0.25373143
metab_218	(S)-5-Hydroxymethyl-2[5H]-furanone	C5H6O3	0.6974	pos	M+H	115.0392	1.9353528670284
metab_219	2-methylpyridin-3-ol	C6H7NO	0.6974	pos	M+H	110.0603	2.5251141924464
metab_220	Homoserine	C4H9NO3	0.6974	pos	M+H-2H2O	84.0449	4.6381590424966
metab_221	3-Methyl-2-oxobutanoic acid	C5H8O3	0.6791	pos	M+H	117.0548	1.7605899695548
metab_222	2-Pyrrolidinecarboxylic acid	C5H9NO2	0.6215	pos	M+H	116.0709	2.3045431941316
metab_223	Maltol	C6H6O3	0.6029	pos	M+H	127.039	0.39822252912627
metab_224	13-OxoODE	C18H30O3	9.7954	pos	M+H	295.2266	-0.477053084
metab_225	3-Methylbenzaldehyde	C8H8O	3.3095	pos	M+H	121.065	1.9965288304369
metab_226	4-hydroxybutanoic acid	C4H8O3	0.5848	pos	M+H-H2O	87.0446	4.8751348670484
metab_227	9(S)-HOTrE	C18H30O3	13.4438	pos	M+H	295.2267	-0.293064481
metab_228	Adenosine	C10H13N5O4	1.0056	pos	M+H	268.104	0.027957573984682
metab_230	Atractylodin	C13H10O	6.8046	pos	M+H	183.0803	-1.044191924
metab_231	Beta-Sitostenone	C29H48O	13.8525	pos	M+H	413.3776	-0.561752414
metab_232	Betaine	C5H11NO2	0.6029	pos	M+H	118.0863	0.71559658656919
metab_234	Catechol	C6H6O2	1.0485	pos	M+H	111.0444	2.8048870404967
metab_235	Farrerol	C17H16O5	7.9237	pos	M+H	301.1069	-0.381125942
metab_236	Hydnocarpic acid	C16H28O2	8.9729	pos	M+H	253.2161	-0.567265463
metab_237	Kainic acid	C10H15NO4	3.732	pos	M+H-H2O	196.097	0.80198987827489
metab_238	Lawsone	C10H6O3	4.4218	pos	M+H	175.0391	0.60069673840888
metab_239	Ligustilide	C12H14O2	5.4809	pos	M+H	191.1066	-0.106286857
metab_240	Lycorine	C16H17NO4	7.2608	pos	M+H	288.1229	-0.494001154
metab_241	Medicarpin	C16H14O4	8.312	pos	M+H	271.0963	-0.717774884
metab_242	Menthofuran	C10H14O	14.2683	pos	M+H-H2O	133.1013	0.70838501605729
metab_243	Methyl cinnamate	C10H10O2	6.6216	pos	M+H	163.0753	-0.141588134

NO.	Metabolite	Formula	Retention time	Mode	Adducts	m/z	Mass Error (ppm)
metab_244	Nicotinamide	C6H6N2O	0.6974	pos	M+H	123.0555	1.3573195275734
metab_245	Oleamide	C18H35NO	13.2856	pos	M+H	282.279	-0.457076624
metab_246	P-Tolualdehyde	C8H8O	1.0263	pos	M+H	121.065	2.042355305
metab_247	Phenol	C6H6O	4.1997	pos	M+NH4	112.0759	2.7080470571598
metab_248	Phosphate	H3O4P	5.2147	pos	M+ACN+H	140.0107	0.18369903948415
metab_251	Vernolic acid	C18H32O3	12.2837	pos	M+H-H2O	279.2318	-0.306515433
metab_252	Vindoline	C25H32N2O6	3.3988	pos	M+NH4	474.26	0.23199356025071
metab_253	D-(+)-Galactose	C6H12O6	0.6133	neg	M-H	179.0553	-4.683366736
metab_254	D-(-)-Ribose	C5H10O5	0.6779	neg	M-H2O-H	131.0338	-8.020191234
metab_255	D-ribonolactone	C5H8O5	0.6981	neg	M-H	147.0288	-7.52921735
metab_257	Uridine	C9H12N2O6	1.0086	neg	M-H	243.062	-0.860230534
metab_259	Homogentisic acid	C8H8O4	2.2229	neg	M-H	167.0341	-5.326311735
metab_261	4-pyridoxic acid	C8H9NO4	2.3524	neg	M-H	182.0451	-4.199818572
metab_262	5-Hydroxyferulic acid methyl ester	C10H10O5	2.3524	neg	M+Hac-H	269.0669	0.72762916983781
metab_263	C-veratroylglycol	C10H12O5	2.6708	neg	M-H	211.0608	-2.031137081
metab_264	Acetovanillone	C9H10O3	2.6936	neg	M-H	165.0547	-5.954678002
metab_265	1-Methoxy-3-carbaldehyde	C10H9NO2	2.7387	neg	M+Hac-H	234.0769	-1.916969549
metab_266	4-Hydroxybenzoic acid glucoside	C13H16O8	2.7387	neg	M-H	299.0774	0.36823764293998
metab_268	Ketoleucine	C6H10O3	2.7844	neg	M+Hac-H	189.0761	-5.898007538
metab_269	2-Isopropylmalic acid	C7H12O5	3.1663	neg	M-H	175.0603	-4.900598286
metab_270	Esculetin	C9H6O4	3.347	neg	M-H	177.0185	-4.512088746
metab_271	3',4'-Dihydroxyacetophenone	C8H8O3	3.3927	neg	M-H	151.039	-6.752376089
metab_272	Feruloyl hexoside	C16H20O9	3.4611	neg	M-H	355.1037	0.74521025670038
metab_273	Gamma-glu-leu	C11H20N2O5	3.7994	neg	M-H2O-H	241.1192	-0.63896456

NO.	Metabolite	Formula	Retention time	Mode	Adducts	m/z	Mass Error (ppm)
metab_274	2-Hydroxycaproic acid	C6H12O3	3.8674	neg	M-H	131.0702	-8.714050147
metab_275	3-hydroxycoumarin	C9H6O3	3.8902	neg	M-H	161.0235	-5.901745805
metab_276	2,4-Dihydroxycinnamic acid	C9H8O4	3.9129	neg	M+Hac-H	239.0559	-1.477611495
metab_277	3'-Hydroxyacetophenone	C8H8O2	4.0233	neg	M-H	135.044	-8.767695367
metab_278	5-Acetylsalicylic acid	C9H8O4	4.0901	neg	M-H	179.0341	-4.674252823
metab_279	Cleroindicin B	C8H14O3	4.1129	neg	M+Hac-H	217.1077	-3.408893814
metab_280	Ethyl gallate	C9H10O5	4.1357	neg	M-H	197.0448	-3.527742179
metab_281	Heptanoic acid	C7H14O2	4.1803	neg	M-H2O-H	111.0802	-9.933085858
metab_282	Syringaldehyde	C9H10O4	4.2031	neg	M-H	181.0498	-4.37929278
metab_283	Dihydroferulic acid	C10H12O4	4.2031	neg	M-H	195.0654	-4.612889799
metab_284	7-hydroxycoumarine	C9H6O3	4.2259	neg	M-H	161.0235	-5.79484992
metab_285	Phloretic acid	C9H10O3	4.2259	neg	M+Hac-H	225.0764	-3.182883609
metab_287	P-Hydroxy-cinnamic acid	C9H8O3	4.2914	neg	M+Na-2H	185.0212	-4.86998786
metab_288	2-methoxy-4-vinylphenol	C9H10O2	4.314	neg	M-H	149.0597	-7.217799055
metab_289	4-Mthoxysalicylaldehyde	C8H8O3	4.4044	neg	M-H	151.0391	-6.689394352
metab_290	N-trans-Feruloyloctopamine	C18H19NO5	4.4273	neg	M-H	328.1194	1.0577154180173
metab_291	Vanillyl alcohol	C8H10O3	4.4952	neg	M-H	153.0547	-6.634366664
metab_292	2,3-Dihydroxytoluene	C7H8O2	4.518	neg	M-H	123.0439	-9.831372636
metab_293	Phellodenol B	C11H10O4	4.5408	neg	M-H	205.0501	-2.577164241
metab_294	Fa(9:0)	C9H18O2	4.5636	neg	M+FA-H	203.1283	-3.474147665
metab_295	4-Methylbenzoic Acid	C8H8O2	4.5863	neg	M-H	135.044	-8.215417395
metab_296	5,7-Dihydroxychromone	C9H6O4	4.5863	neg	M-H	177.0185	-4.728189514
metab_297	4-Methoxyphenylacetic acid	C9H10O3	4.6091	neg	M-H	165.0548	-5.649868233
metab_298	Trans-2-Octenal	C8H14O	4.6997	neg	M-H	125.096	-9.605762051

NO.	Metabolite	Formula	Retention time	Mode	Adducts	m/z	Mass Error (ppm)
metab_299	N-Caffeoyltyramine	C17H17NO4	4.7645	neg	M-H	298.1087	0.72866242723053
metab_300	2,6-Dimethoxybenzoquinone	C8H8O4	4.8679	neg	M-H	167.0341	-5.413861374
metab_301	3,4-Dimethylbenzoic acid	C9H10O2	5.0277	neg	M-H	149.0597	-7.103605702
metab_302	(+)-Nortrachelogenin	C20H22O7	5.0679	neg	M+Hac-H	433.1507	0.77201475004263
metab_303	2',6'-dihydroxy-4'-methoxyacetophenone	C9H10O4	5.1989	neg	M-H	181.0498	-4.461584735
metab_304	Tyrosol	C8H10O2	5.5491	neg	M-H	137.0597	-8.181650966
metab_305	N-trans-Feruloyltyramine	C18H19NO4	5.5717	neg	M-H	312.1245	1.0340222560079
metab_306	10-hydroxy-2e-decenoic acid	C10H18O3	5.6508	neg	M+Hac-H	245.1394	-0.648175659
metab_307	9-oxononanoic acid	C9H16O3	7.0819	neg	M-H	171.1018	-5.074678182
metab_308	Corchorifatty acid F	C18H32O5	7.6967	neg	M-H	327.218	0.91594723000352
metab_309	Ethyl p-Coumarate	C11H12O3	8.0672	neg	M-H	191.0707	-3.56635643
metab_310	9,12,13-Todea	C18H34O5	8.5759	neg	M-H	329.2336	0.78856250417088
metab_311	Grossamide	C36H36N2O8	8.7227	neg	M-H	623.2408	1.4663751873463
metab_312	Tianshic acid	C18H34O5	8.8324	neg	M-H	329.2336	0.88652096847991
metab_313	Prostaglandin a1	C20H32O4	9.3418	neg	M-H	335.2232	1.3533607281752
metab_314	Prostaglandin B1	C20H32O4	12.4657	neg	M+FA-H	381.2262	-6.032980611
metab_315	(3R)-3-hydroxydodecanoic acid	C12H24O3	12.8147	neg	M-H	215.1648	-2.360730566
metab_316	Civetone	C17H30O	13.8494	neg	M+FA-H	295.2279	0.15138612676736
metab_317	2-hydroxyhexadecanoic acid	C16H32O3	14.1168	neg	M-H	271.2281	0.69838444847402
metab_318	Chemanox 22	C25H36O2	14.279	neg	M-H	367.2644	0.36843434320279
metab_319	Kukoamine B	C28H42N4O6	13.535	neg	M-H	529.3022	-1.794460312
metab_320	Coronaric acid	C18H32O3	13.2117	neg	M-H	295.228	0.28248530414407
metab_321	2-(2-ethylhexoxycarbonyl)benzoic acid	C16H22O4	12.9908	neg	M-H	277.1446	0.27791685373999
metab_322	Methyl hexadecanoate	C17H34O2	12.7683	neg	M+FA-H	315.2542	0.47026948549622

NO.	Metabolite	Formula	Retention time	Mode	Adducts	m/z	Mass Error (ppm)
metab_323	5-O-Methylembelin	C18H28O4	8.4003	neg	M-H	307.1918	1.0286625154461
metab_324	Traumatic acid	C12H20O4	8.3455	neg	M-H	227.1285	-1.468009245
metab_325	Undecanedioic acid	C11H20O4	7.3634	neg	M-H	215.1285	-1.882949289
metab_326	3-hydroxy-1-(4-hydroxyphenyl)propan-1-one	C9H10O3	6.4966	neg	M-H2O-H	147.0441	-6.387945772
metab_327	Tuberonic acid	C12H18O4	6.4808	neg	M-H	225.1129	-1.494880595
metab_328	Ethyl caffeate	C11H12O4	6.4666	neg	M-H	207.0658	-2.555194795
metab_329	Methyl 4-hydroxycinnamate	C10H10O3	6.3607	neg	M-H	177.0549	-4.598233292
metab_330	Moracin M	C14H10O4	6.0833	neg	M-H	241.0505	-0.494808128
metab_331	Sebacic acid	C10H18O4	6.0109	neg	M-H	201.1126	-2.961950011
metab_332	2-formamidobenzoic acid	C8H7NO3	5.8736	neg	M-H	164.0344	-5.50746231
metab_333	Eugenin	C11H10O4	5.6701	neg	M-H	205.0501	-2.731087296
metab_334	(-)-Senkyunolide J	C12H18O4	5.4822	neg	M-H	225.1129	-1.423262095
metab_335	Epomediol	C10H18O3	5.2217	neg	M+FA-H	231.1235	-1.69485334
metab_336	3-Hydroxybenzoic Acid	C7H6O3	5.1763	neg	M-H	137.0233	-8.305180281
metab_337	9-Hydroxynonanoic acid	C9H18O3	5.1136	neg	M-H	173.1175	-4.867566034
metab_338	Azelaic acid	C9H16O4	4.9597	neg	M-H	187.0967	-4.479038587
metab_339	Caprylic acid	C8H16O2	4.9597	neg	M-H2O-H	125.0959	-8.915668182
metab_340	Isopeonol	C9H10O3	4.8083	neg	M-H	165.0548	-5.679087643
metab_341	Jioglutin E	C11H20O5	4.7855	neg	M-H	231.1235	-1.086432106
metab_342	Eseramine	C16H22N4O3	4.7452	neg	M-H	317.1608	-3.345446584
metab_343	Isomucronulatol	C17H18O5	4.6318	neg	M+FA-H	347.1117	-6.514620697
metab_344	Epinephrine	C9H13NO3	4.6091	neg	M-H2O-H	164.0708	-4.962495062
metab_345	Hematoxylin	C16H14O6	4.5408	neg	M+FA-H	347.0775	0.890630495
metab_346	N-feruloyloctopamine	C18H19NO5	4.4273	neg	M-H2O-H	310.1088	0.93499837001783

NO.	Metabolite	Formula	Retention time	Mode	Adducts	m/z	Mass Error (ppm)
metab_347	Fraxinol	C11H10O5	4.4273	neg	M-H	221.0451	-1.838086195
metab_348	L-Tryptophan	C11H12N2O2	4.4273	neg	M-H	203.0821	-2.577053444
metab_349	Indole-3-acetic acid	C10H9NO2	4.314	neg	M-H	174.0552	-4.982470056
metab_350	Isoscopoletin	C10H8O4	4.2914	neg	M-H	191.0342	-3.9449704
metab_351	Octopamine, N-p-coumaroyl-	C17H17NO4	4.2484	neg	M-H	298.1086	0.30585187727532
metab_352	Suberic acid	C8H14O4	4.1803	neg	M-H	173.0811	-4.843066767
metab_353	Glu-Phe	C14H18N2O5	4.0901	neg	M-H2O-H	275.104	0.93993475618922
metab_354	3-hydroxycinnamic acid	C9H8O3	4.0016	neg	M-H	163.039	-6.279081988
metab_355	2,6-dihydroxybenzoic acid	C7H6O4	4.0016	neg	M-H	153.0183	-6.805783366
metab_356	7,8-Dihydroxycoumarin	C9H6O4	3.9357	neg	M+Hac-H	237.0402	-1.801272495
metab_357	Salvianic acid A	C9H10O5	3.8674	neg	M-H2O-H	179.0342	-4.031790383
metab_358	Vanillin	C8H8O3	3.8449	neg	M-H	151.039	-6.84558562
metab_359	Phloracetophenone	C8H8O4	3.7994	neg	M-H	167.0341	-5.411385856
metab_360	Fraxetin	C10H8O5	3.7766	neg	M-H	207.0294	-2.54915017
metab_361	Piperonal	C8H6O3	3.7539	neg	M-H	149.0234	-6.968860087
metab_362	Plumbagin	C11H8O3	3.7311	neg	M-H	187.0394	-3.745708915
metab_363	Sinapate	C11H12O5	3.6402	neg	M-H	223.0607	-2.116920593
metab_364	Icariside F2	C18H26O10	3.5287	neg	M-H	401.1456	0.63329025842045
metab_365	3,4-dihydroxyphenylacetic acid	C8H8O4	3.5063	neg	M-H2O-H	149.0233	-6.498548571
metab_366	Isoeugenitol	C11H10O4	3.4383	neg	M-H	205.0501	-2.685497672
metab_367	Pimelic acid	C7H12O4	3.3927	neg	M-H	159.0653	-6.04567171
metab_368	Vanillic acid	C8H8O4	3.347	neg	M-H	167.0341	-5.495286542
metab_369	3,5-dimethoxy-9,10-dihydrophenanthrene-2,7-diol	C16H16O4	3.3243	neg	M-H	271.0978	0.83323608370188
metab_370	5-Hydroxyferulate	C10H10O5	3.2116	neg	M-H	209.045	-2.684553961

NO.	Metabolite	Formula	Retention time	Mode	Adducts	m/z	Mass Error (ppm)
metab_371	Homovanillic acid	C9H10O4	3.1663	neg	M-H	181.0498	-4.354410639
metab_372	Epicatechin	C15H14O6	3.1216	neg	M-H	289.0719	0.64177049348489
metab_373	Caffeic acid hexoside	C15H18O9	3.0544	neg	M-H	341.0878	0.049150284765291
metab_374	Patulin	C7H6O4	3.0318	neg	M-H	153.0183	-6.894980082
metab_375	4-hydroxy-benzoate	C7H6O3	2.9876	neg	M-H	137.0233	-8.398078832
metab_376	Isophthalic acid	C8H6O4	2.9431	neg	M-H	165.0184	-5.859226556
metab_377	2-methoxy-4-methylphenol	C8H10O2	2.9431	neg	M-H	137.0595	-9.348593308
metab_378	Regaloside B	C20H26O11	2.8979	neg	M+FA-H	487.1461	0.88470098427685
metab_379	4-Methyl-6,7-dihydroxycoumarin	C10H8O4	2.8526	neg	M+Hac-H	251.0561	-0.565560711
metab_380	P-Anisic Acid	C8H8O3	2.7387	neg	M-H	151.039	-6.946174072
metab_381	Coumaroyl hexoside	C15H18O8	2.716	neg	M-H	325.0931	0.68190506600249
metab_382	Piperonylic acid	C8H6O4	2.3978	neg	M+Hac-H	225.0401	-2.408704237
metab_383	Protocatechuic acid	C7H6O4	2.3978	neg	M-H	153.0183	-6.632460336
metab_384	Pyridoxine	C8H11NO3	2.3296	neg	M-H	168.0657	-5.377617146
metab_385	2-methylsuccinic acid	C5H8O4	2.2614	neg	M-H	131.0338	-8.746120509
metab_386	Thymidine	C10H14N2O5	2.0899	neg	M-H	241.0828	-0.814781964
metab_387	L-(-)-3-Phenyllactic acid	C9H10O3	1.9533	neg	M-H	165.0548	-5.698043364
metab_393	Trehalose	C12H22O11	0.6355	neg	M-H	341.1089	-0.046712743
metab_394	Sucrose	C12H22O11	0.6133	neg	M+Cl	377.0859	0.7217630616981
metab_395	1-O-Feruloyl-beta-D-glucose	C16H20O9	2.9431	neg	M-H	355.1037	0.60830023288113
metab_396	9,10-Dihydroxystearate	C18H36O4	12.9107	neg	M-H	315.2542	0.32216457252972
metab_397	Abscisic acid	C15H20O4	5.3663	neg	M-H	263.1291	0.69178764088638
metab_398	Caffeic acid	C9H8O4	3.3927	neg	M-H	179.0342	-4.491841394
metab_399	Geraniol	C10H18O	5.005	neg	M+FA-H	199.1333	-4.090381554
metab_401	Isoferulic acid	C10H10O4	3.4611	neg	M-H	193.05	-3.468859578
metab_402	Isosakuranetin	C16H14O5	4.9821	neg	M+FA-H	331.0826	1.0274524617305

NO.	Metabolite	Formula	Retention time	Mode	Adducts	m/z	Mass Error (ppm)
metab_403	L-Arabinose	C5H10O5	0.5929	neg	M-H	149.0445	-7.18945916
metab_404	Melilotoside	C15H18O8	0.7164	neg	M-H	325.0932	0.9397193112488
metab_405	Pantothenic acid	C9H17NO5	2.3978	neg	M-H	218.1029	-2.043732479
metab_406	Piceatannol	C14H12O4	4.8311	neg	M-H	243.0662	-0.416788347
metab_407	Sorbose	C6H12O6	0.6779	neg	M-H2O-H	161.0446	-5.525029942
metab_408	Sweroside	C16H22O9	3.234	neg	M-H	357.1194	0.8229220903604
