# Supplementary material for: Ameliorative Effect of Erjing Pills on Retinal Damage in Rats with Diabetic Retinopathy
Source: Pharmaceuticals (Basel). 2026 Jun 15;19(6):940. doi: 10.3390/ph19060940 (PMC13305654; doi:10.3390/ph19060940)
Supplement: Supplementary file 1 [file pharmaceuticals-19-00940-s001.zip › TableS2.pdf]

Table S2. Key operating parameters of the Q Exactive Orbitrap mass spectrometer

Parameter	Value / Setting
**Ion Source Conditions**	
Sheath Gas Flow Rate	50 arb
Auxiliary Gas Flow Rate	13 arb
Heater Temperature	450 °C
Capillary Temperature	320 °C
S-Lens RF Level	40
Spray Voltage (Positive)	3500 V
Spray Voltage (Negative)	-3000 V
**Full MS Scan**	
Scan Range	m/z 70–1050
Resolution (at m/z 200)	70,000
AGC Target	3×10^6
Maximum Injection Time	100 ms
Data-Dependent MS/MS	
Resolution (at m/z 200)	17,500
Loop Count (Top N)	10
Stepped Normalized Collision Energy (NCE)	20%, 40%, 60%
